# Chitosan/β-cyclodextrin nanocarriers enhance 5-fluorouracil efficacy against colorectal cancer via pH-responsive release and apoptosis modulation

**DOI:** 10.1038/s41598-026-60468-z

**Published:** 2026-07-07

**Authors:** Amr M. A. Mohamed, Hemat M. Dardeer, Ali M. A. Ahmed, M. Yasser Alsedfy, Amr E. Abd Elhadi, Abdelmoniem M. A. Elsanosy, Abdelhakam Esmaeil Mohamed Ahmed, Alaa Hassan Said

**Affiliations:** 1 Department of Zoology, Faculty of Science, Qena University, Qena, 83523 Egypt; 2 Department of Chemistry, Faculty of Science, Qena University, Qena, 83523 Egypt; 3 Molecular Biotechnology Program, Faculty of Science, Qena University, Qena, 83523 Egypt; 4 Electronics and Nano Devices lab, Faculty of Science, Qena University, Qena, 83523 Egypt; 5 Radiology Department, Applied Health Sciences Faculty, Sphinx University, New Assiut, Egypt; 6 Faculty of Agriculture, Food Sciences and Environmental Management, Institute of Food Science, University of Debrecen, Böszörményi str. 138, Debrecen, 4032 Hungary; 7 Faculty of Forestry, University of Khartoum, Khartoum North, 13314 Sudan

**Keywords:** In vitro colorectal cancer, 5-fluorouracil, Chitosan, β-cyclodextrin-Nanocarrier, Gene expression, In silico, Protein–protein interaction (PPI) network., Biochemistry, Biotechnology, Cancer, Drug discovery

## Abstract

Globally, colorectal cancer continues to be a major cause of cancer-related death. Clinical application of 5‑fluorouracil (5‑FU) is constrained by reduced bioavailability and dose‑limiting toxicity. To overcome these restrictions, a ternary drug delivery system was developed in this study. Chitosan and β‑cyclodextrin were physically assembled into a pseudopolyrotaxane complex, and 5‑FU was incorporated by solvent evaporation to form a solid dispersion. The resulting nanocomposite had a mean hydrodynamic diameter of 187 nm and a zeta potential of + 32.5 mV. Fourier transform infrared spectroscopy revealed peak shifts consistent with non‑covalent interactions. X‑ray diffraction confirmed amorphization of 5‑FU within the formulation. The ternary system released 5‑FU more rapidly at pH 5.5 than at pH 7.4. In HCT‑116 colorectal cancer cells, the 5‑FU/chitosan/β‑cyclodextrin formulation showed an IC_50_ of 16.5 µM, compared with 42.5 µM for free 5‑FU and 27.5 µM for the binary 5‑FU/β‑cyclodextrin complex. Gene expression analysis indicated upregulation of *P53* and *Caspase‑3* and downregulation of *BCL2* and *VEGF*. A complementary in silico analysis of 5-FU-responsive genes (from public dataset GSE183977) identified a protein‑protein interaction network enriched in apoptosis and cell‑cycle pathways, with *IL6*, *MYC*, *EGR1*, and *ATF3* as central hub genes. The obtained results show that incorporation of 5‑FU into a chitosan/β‑cyclodextrin matrix improves its activity against colorectal cancer cells. This effect is attributed to enhanced solubility, pH‑responsive release, and modulation of apoptotic and angiogenic pathways.

## Introduction

Colorectal cancer (CRC) is ranked second in terms of cancer-related mortality and third in terms of global cancer incidence, with GLOBOCAN 2020 recording approximately 1.93 million new cases and 930,000 deaths, roughly 10% of the worldwide cancer burden^1^. Projections to 2040 anticipate a 63% rise in incidence and a 73% rise in mortality, driven largely by dietary, lifestyle, and inflammatory risk factors^2^.

Systemic toxicity, poor drug absorption, and chemoresistance make treatment outcomes less than ideal in advanced stages. This arises despite improvements in chemotherapy, radiation, and surgery^3^. 5-Fluorouracil (5-FU) is the primary chemotherapy used in CRC treatment. It functions as a thymidylate synthase inhibitor, disrupting the synthesis of DNA and RNA^4^. However, its rapid metabolism, short plasma half-life, and toxicities including myelosuppression and mucositis restrict its therapeutic application^5^. Drug resistance from improved DNA repair systems and anti-apoptotic signaling further limits 5-FU effectiveness^6^.

Nanoparticulate drug delivery methods have addressed some of these limitations. These systems extend circulation time, decrease systemic side effects, and enhance drug stability.

The enhanced permeability and retention (EPR) effect facilitates targeting^7^. Biopolymer-based carriers have demonstrated promise among these systems. Chitosan and β-cyclodextrin (β-CD) offer functionalization potential, biodegradability, and biocompatibility^8^.

Chitosan is a cationic polysaccharide obtained from the deacetylation of chitin, was selected for its dual functional advantage: its protonated amine groups confer pH-responsive swelling in the acidic tumor microenvironment, and its positive surface charge enhances electrostatic interaction with cell membranes, promoting adsorptive endocytosis and improving mucoadhesion^9^.

β-Cyclodextrin is a cyclic oligosaccharide containing seven cone-shaped glucopyranose units. Its hydrophobic core forms inclusion complexes with various drugs^10^. Encapsulation of 5-FU in β-CD stabilizes the drug against hydrolysis and enzymatic destruction while allowing regulated release^11^. Integrating β-CD with chitosan in hybrid nanocarriers combines improved stability, mucoadhesion, drug loading, and pH-responsive release^12^. Chitosan/cellulose fiber bionanocomposites loaded with 5-FU demonstrated specific cytotoxicity against HCT-116 cells with encapsulation efficiency of approximately 86%^13^.

Network pharmacology integrates differential gene expression data with curated protein–protein interaction databases to identify how treatment-induced transcriptional changes propagate through cellular signaling networks^14^. By mapping hub genes and enriched functional modules, this approach translates cellular gene expression data into mechanistic hypotheses about drug resistance and apoptotic signaling, complementing in vitro cytotoxicity and RT-qPCR data with a systems-level perspective. For 5-FU specifically, network-based analysis may reveal how nanocarrier-mediated delivery alters resistance-associated gene interactions beyond what single-gene assays can detect.

This study developed a ternary 5-FU/chitosan/β-cyclodextrin nanocarrier and evaluated its anticancer activity against HCT-116 colorectal cancer cells through parallel in vitro and in silico approaches. The nanocarrier was characterized by FTIR, XRD, DLS, and SEM. Cytotoxicity was quantified by SRB assay, and expression of *P53*, *Caspase-3*, *BCL2*, and *VEGF* was measured by RT-qPCR at IC_50_ equivalent concentrations. PPI network analysis and functional enrichment of 5-FU-responsive genes from a public transcriptomic dataset were used to provide systems-level context for the observed cellular responses.

## Materials and methods

### Materials

5-Fluorouracil (≥ 99%, HPLC grade) was purchased from Damas-Beta Co., China, and β-Cyclodextrin (≥ 98%, molecular weight 1135 g/mol) from Merck Co., Germany. Chitosan (medium molecular weight, 190–310 kDa, degree of deacetylation 75–85%, viscosity 200–800 cP) was obtained from Merck Co., Germany. N, N-Dimethylformamide (anhydrous, 99.8%), glacial acetic acid (≥ 99.7%), and all other reagents were of analytical grade. Cell line of human colorectal cancer HCT-116 was obtained from Nawah Scientific Inc. (Cairo, Egypt). RPMI-1640 medium, fetal bovine serum, penicillin–streptomycin solution, and trypsin-EDTA were purchased from Gibco (Thermo Fisher Scientific, USA).

### Preparation of formulations

#### Preparation of 5-FU/β-CD

A physical mixture of 5-FU and β-CD was prepared at the same weight ratio used for the inclusion complex (1:50, w/w). The components were gently ground in a mortar for 10 min to ensure homogeneous mixing without inducing complex formation.

#### Preparation of 5-FU/β-CD inclusion complex

**Fig. 1:**
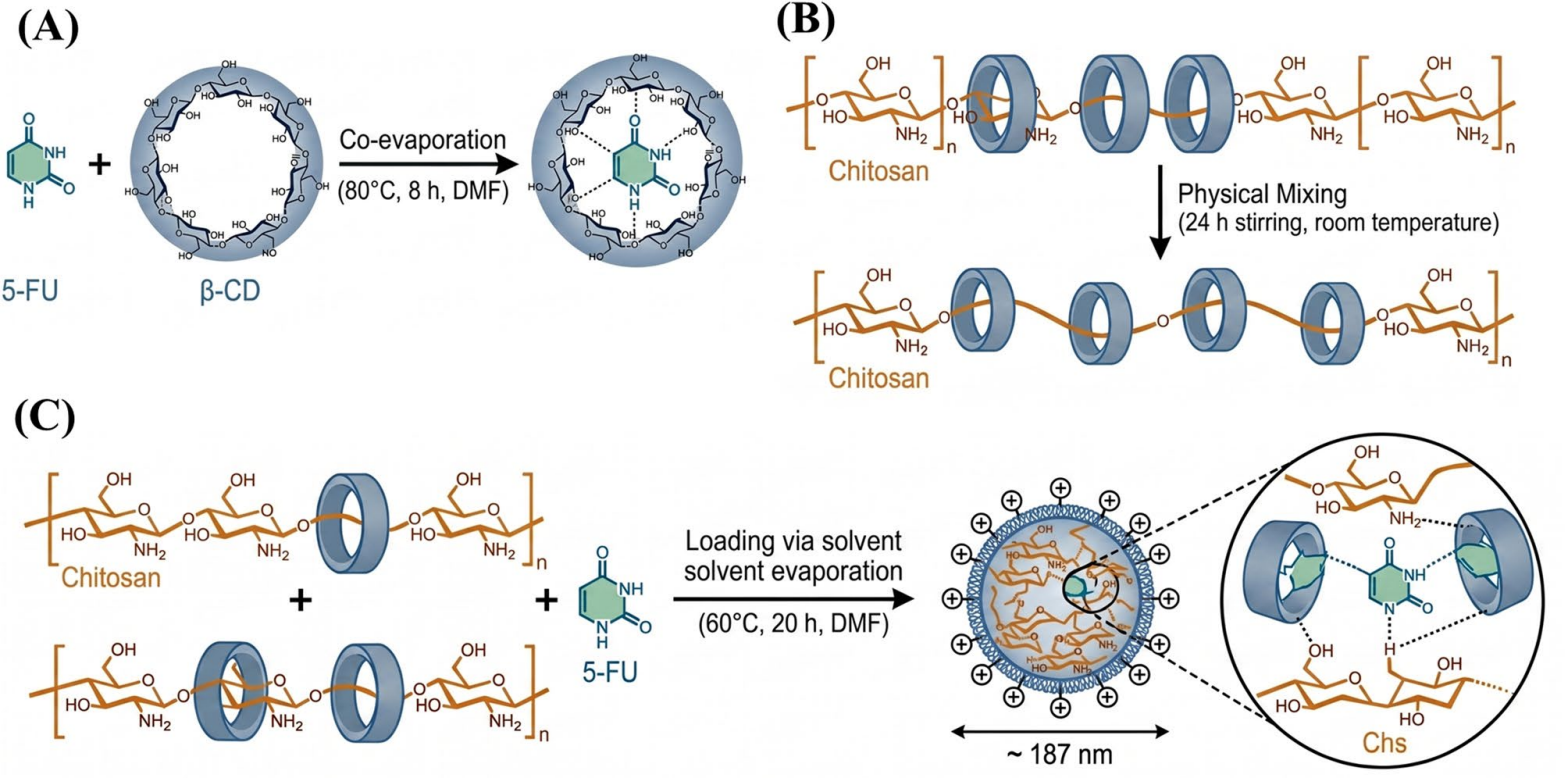
Schematic illustration of the preparation of (**A**) 5-fluorouracil/β-cyclodextrin (5-FU/β-CD) via co-evaporation method, (**B**) chitosan/β-cyclodextrin (Chs/β-CD) pseudopolyrotaxane complex via physical mixing method and (**C**) ternary 5-fluorouracil/chitosan/β-cyclodextrin (5-FU/Chs/β-CD) formulation via solvent evaporation/co-precipitation method. Created with BioRender.com .

A co-evaporation method was used to obtain the 5-FU/β-CD inclusion complex^15^. Under magnetic stirring, 5-FU (0.04 g) was dissolved in 30 mL of DMF. β-CD (2 g) was gradually added. The suspension was maintained under continuous stirring at 80 °C for 8 h. After being transferred into sterile Petri dishes, the resultant solution was allowed to dry at room temperature for seven days. The dried solid was scraped, gently pulverized, and stored in airtight containers at room temperature. A schematic representation is shown in Fig. 1A.

#### Preparation of Chitosan/β-CD Pseudopolyrotaxane Complex

Physical mixing was used to synthesize the chitosan/β-CD pseudopolyrotaxane complex^15^. A continuous stirring process at 600 rpm was used to dissolve β-CD (1.1 g) in 50 mL of distilled water at 50 °C. The solution was cooled to room temperature. After dissolving 0.25 g of chitosan in 50 mL of 1% (v/v) aqueous acetic acid, the mixture was stirred for 12 h. At room temperature, the β-CD solution was stirred at 600 rpm while the chitosan solution was added dropwise (1 mL/min).

The mixture was stirred for an additional 24 h. An opaque, milky-white suspension formed. The precipitate was collected by centrifugation at 12,000 × g for 20 min. Washing was performed three times with distilled water and once with 0.1 M ammonium hydroxide. The final product was lyophilized for 48 h and stored in a desiccator (Fig. 1B**)**.

#### Preparation of 5-FU/Chitosan/β-CD Ternary Formulation

The ternary formulation (5-FU/Chs/β-CD) was prepared using solvent evaporation^16,17^. The pre-formed Chs/β-CD pseudopolyrotaxane complex (0.8 g) was dispersed in 30 mL of anhydrous DMF at 60 °C for 2 h under magnetic stirring at 600 rpm. 5-FU (0.5 g) was dissolved in 10 mL of anhydrous DMF with brief sonication (5 min). At room temperature, the 5-FU solution was added dropwise (1 mL/min) to the Chs/β-CD dispersion. For 20 h, the mixture was heated to 60 °C and stirred, forming a fin white precipitate. Vacuum filtration (sintered glass funnel, porosity #4) was used to collect the precipitate. Washing was performed three times with 50 mL of distilled water. After being air-dried for 24 h at room temperature, the product was further dried for 48 h over silica gel in a vacuum desiccator. The 5-FU/Chs/β-CD powder was stored in sealed amber glass vials at 4 °C (Fig. 1C**)**.

### Characterization of formulations

#### Particle size and zeta potential analysis

Hydrodynamic diameter, polydispersity index (PDI), and zeta potential of the 5-FU/Chs/β-CD ternary formulation were determined by dynamic light scattering (DLS) using a Zetasizer Nano ZS (Malvern Instruments, UK). Prior to testing, samples were dispersed at a concentration of 1 mg/mL in deionized water and sonicated for 10 min. At 25 °C and a scattering angle of 173°, measurements were conducted in triplicate. The findings are presented as mean ± standard deviation (SD).

#### Fourier transform infrared spectroscopy (FTIR)

FTIR spectra were recorded using a Jasco Model 4100 spectrometer (Japan) in the wavenumber range of 4000–400 cm^− 1^ with a resolution of 4 cm^− 1^. Samples were prepared as KBr pellets. Spectra were obtained for pure 5-FU, pure β-CD, pure chitosan, the physical mixture (5-FU + β-CD), the binary 5-FU/β-CD inclusion complex, and the ternary 5-FU/Chs/β-CD formulation.

#### X-ray diffraction (XRD)

X-ray diffraction patterns were obtained using a Bruker D8 Advance diffractometer (Germany) with Cu Kα radiation (λ = 1.5406 Å) operating at 40 kV and 40 mA. Samples were scanned over a 2θ range of 5–80° with a step size of 0.02° and a scan speed of 2°/min.

#### Scanning electron microscopy (SEM)

Surface morphology was examined using a Zeiss Sigma 500 VP FESEM (Germany). Samples were dispersed in deionized water at 0.1 mg/mL, drop-cast onto aluminum stubs, and dried under vacuum. A thin gold layer was sputter-coated to enhance conductivity. Images were acquired at an accelerating voltage of 5 kV with magnifications of 10,000× and 50,000×.

#### In vitro drug release study

A dialysis bag diffusion method was used to assess the release profile of 5-FU from the ternary formulation^18^. The 5-FU/Chs/β-CD formulation (10 mg, equivalent to approximately 2.5 mg of 5-FU) was dispersed in 2 mL of release medium and placed in a dialysis bag (MWCO 12–14 kDa). The bag was continuously shaken at 100 rpm while immersed in 50 mL of release media at 37 °C. Two release media were employed: acetate buffer (pH 5.5) and phosphate-buffered saline (PBS, pH 7.4). Aliquots of 1 mL were removed and replaced with new media at predetermined time interval (0.5, 1, 2, 4, 8, 12, 24, 48, and 72 h). UV-Vis spectrophotometry at 266 nm was used to measure the amount of released 5-FU in triplicate.

#### Drug loading content and encapsulation efficiency

The ternary formulation’s encapsulation efficiency (EE) and drug loading content (DLC) were calculated. The 5-FU/Chs/β-CD powder (5 mg) was dissolved in 10 mL of PBS (pH 7.4) under sonication for 30 min. A membrane filter with a 0.45 μm pore size was used to filter the solution. Using UV-Vis spectrophotometry at 266 nm, the concentration of 5-FU was measured. DLC and EE were calculated using standard **equations**^17^.

1 $$\:\mathrm{D}\mathrm{L}\mathrm{C}\:\left({\%}\right)=\frac{\mathrm{W}\mathrm{e}\mathrm{i}\mathrm{g}\mathrm{h}\mathrm{t}\:\mathrm{o}\mathrm{f}\:\mathrm{d}\mathrm{r}\mathrm{u}\mathrm{g}\:\mathrm{i}\mathrm{n}\:\mathrm{f}\mathrm{o}\mathrm{r}\mathrm{m}\mathrm{u}\mathrm{l}\mathrm{a}\mathrm{t}\mathrm{i}\mathrm{o}\mathrm{n}\:}{\mathrm{W}\mathrm{e}\mathrm{i}\mathrm{g}\mathrm{h}\mathrm{t}\:\mathrm{o}\mathrm{f}\:\mathrm{f}\mathrm{o}\mathrm{r}\mathrm{m}\mathrm{u}\mathrm{l}\mathrm{a}\mathrm{t}\mathrm{i}\mathrm{o}\mathrm{n}}\times\:100$$

2 $$\:\mathrm{E}\mathrm{E}\:\left({\%}\right)=\frac{\mathrm{W}\mathrm{e}\mathrm{i}\mathrm{g}\mathrm{h}\mathrm{t}\:\mathrm{o}\mathrm{f}\:\mathrm{d}\mathrm{r}\mathrm{u}\mathrm{g}\:\mathrm{i}\mathrm{n}\:\mathrm{f}\mathrm{o}\mathrm{r}\mathrm{m}\mathrm{u}\mathrm{l}\mathrm{a}\mathrm{t}\mathrm{i}\mathrm{o}\mathrm{n}\:}{\mathrm{W}\mathrm{e}\mathrm{i}\mathrm{g}\mathrm{h}\mathrm{t}\:\mathrm{o}\mathrm{f}\:\mathrm{d}\mathrm{r}\mathrm{u}\mathrm{g}\:\mathrm{i}\mathrm{n}\mathrm{i}\mathrm{t}\mathrm{i}\mathrm{a}\mathrm{l}\mathrm{l}\mathrm{y}\:\mathrm{a}\mathrm{d}\mathrm{d}\mathrm{e}\mathrm{d}}\times\:100$$

#### Phase solubility study

The phase solubility method was used to assess the impact of β-CD and Chs/β-CD on 5-FU aqueous solubility^19^. Excess 5-FU was added to aqueous solutions containing increasing concentrations of β-CD (0–20 mM) or Chs/β-CD pseudopolyrotaxane (0–10 mg/mL) in sealed vials. After 72 h of shaking at 25 °C, suspensions were filtered through 0.45 μm membrane filters and subjected to UV-Vis spectrophotometry analysis at 266 nm. The slope of linear section of the phase solubility diagram’s was used to estimate the apparent stability constant (Ks).

### In vitro cytotoxicity assay

Cytotoxicity of free 5-FU, binary 5-FU/β-CD inclusion complex, and ternary 5-FU/Chs/β-CD formulation against HCT-116 cells was investigated using the Sulforhodamine B (SRB) assay^20^. Cells were seeded at 5 × 10^5^ cells per well in 96-well plates with 100 µL of complete RPMI-1640 medium (10% fetal bovine serum, 100 U/mL penicillin, 100 µg/mL streptomycin). Following a 24 h incubation period at 37 °C in 5% CO2, the medium was replaced with 100 µL of freshly prepared media with different concentrations (0.05–500 µg/mL equivalent 5-FU concentration). Following a 72 h treatment period, cells were fixed for one hour at 4 °C using 150 µL of 10% trichloroacetic acid. Distilled water was used to wash the wells five times. For ten minutes in the dark, cells were stained with 70 µL of 0.4% SRB solution. After three rounds of 1% acetic acid washing, the plates were allowed to dry in air. Bound dye was dissolved in 150 µL of 10 mM Tris buffer. The absorbance was measured with a TECAN Infinite F50 microplate reader at 540 nm. Cell viability was expressed as a percentage in correlation to the untreated control calculated as^21^:

3 $$\:\text{Cell viability}\:(\%)=\left(\frac{{A}_{\mathrm{sample}}-{A}_{\mathrm{blank}}}{{A}_{\mathrm{control}}-{A}_{\mathrm{blank}}}\right)\times\:100$$

where $\:{A}_{\mathrm{sample}}$, $\:{A}_{\mathrm{control}}$, and $\:{A}_{\mathrm{blank}}$ represent the absorbance of treated wells, untreated control wells, and cell-free medium blanks, respectively. IC₅₀ values were determined by non-linear regression.

### In vitro gene expression analysis (RT-qPCR)

HCT-116 cells were seeded into T25 culture flasks (Greiner Bio-One, Germany) and let to adhere for 24 h under standard culture conditions (37 °C, 5% CO_2_). Cells were divided into four experimental groups. Group 1 (untreated control) received fresh culture medium only. Group 2 was exposed to 42.5 µM free 5-FU. Group 3 was treated with 27.5 µM 5-FU/β-CD inclusion complex. Group 4 received 16.5 µM ternary nanocarrier formulation 5-FU/Chs/β-CD. After 72 h of incubation, cells were harvested by detachment using 0.25% trypsin-EDTA, followed by centrifugation at 200 × g for 5 min at 5 °C.

#### RNA extraction and cDNA synthesis

Total RNA was isolated from 50 mg of each cell pellet using TransZol reagent (Transgen Biotech)^22^. A NanoDrop ND-1000 spectrophotometer (Thermo Scientific) was used to measure the concentration and purity of RNA using 260/280 nm absorbance ratios. cDNA was synthesized using 200 ng of total RNA following the RevertAid First Strand cDNA Synthesis Kit protocol (Thermo Scientific)^23^.

#### Gene expression analysis using qPCR

Quantitative real-time PCR was performed using 2x Maxima SYBR Green Master Mix (Thermo Scientific) on a DT-Lite Real-Time PCR system (DNA Technology). Expression levels of *P53*, *Caspase-3*, *BCL2*, and *VEGF* were evaluated. Relative expression was normalized using *GAPDH* and *β-Actin* as housekeeping genes. The reaction was performed in a total volume of 25 µL contained 12.5 µL 2x Maxima SYBR green qPCR master mix, 1 µL of each primer, 2 µL of cDNA template, and nuclease-free water. Thermal cycling conditions were initiated with initial denaturation at 95 °C for 10 min; followed by 40 cycles of amplification consists of denaturation at 95 °C for 30 s, annealing at temperatures illustrated in Table 1, and extension at 75 °C for 30 s. A final extension step at 95 °C for 5 min was conducted. Melting curve analysis was performed from 70 °C to 90 °C to verify products specify. Samples were analyzed in triplicate. Gene expression levels were evaluated using the comparative Ct (2⁻ΔΔCt) method^24^.

**Table 1:** Primer sequences and annealing temperatures.

Gene	Primer Sequence (5′-3′)	Annealing Temp.	Reference
***GAPDH***	**F**-GTCTCCTCTGACTTCAACAGCG	60 °C	^25^
	**R**-ACCACCCTGTTGCTGTAGCCAA		
***β-Actin***	**F-** AGAAAATCTGGCACCACACC	57 °C	^26^
	**R-** TAGCACAGCCTGGATAGCAA		
***Caspase 3***	**F-**GGAAGCGAATCAATGGACTCTGG	58 °C	^27^
	**R-**GCATCGACATCTGTACCAGACC		
***P53***	**F-**ACCTATGGAAACTACTTCCTGAAA	58 °C	^28^
	**R-**CTGGCATTCTGGGAGCTTCA		
***BCL-2***	**F-**ATGTGTGTGGAGACCGTCAA	56 °C	^29^
	**R-** GCCGTACAGTTCCACAAAGG		
***VEGF***	**F-**CTACCTCCACCATGCCAAGT	56 °C	^30^
	**R-**TCTCTCCTATGTGCTGGCCT		

### In silico network pharmacology and functional enrichment analysis

#### Data retrieval

The Gene Expression Omnibus (GEO) at NCBI (https://www.ncbi.nlm.nih.gov/geo/) was accessed. Keywords “5FU”, “colorectal cancer”, and “fluorouracil” were used. Dataset GSE183977 contained 4 samples in total. Two control samples and two samples of HT29 human CRC cell line treated with 100 µM 5-FU for 2 h were selected.

#### Differentially expressed gene analysis

GEO2R was used to compare gene expressions between sample groups. Genes with absolute log2 fold change > 1.5 and adjusted p-value < 0.05 were considered significantly dysregulated^31^.

#### Protein–protein interaction (PPI) analysis using STRING

Differentially expressed genes (DEGs) were analyzed by constructing PPI networks using the STRING database (https://string-db.org/). Interactions with confidence score > 0.4 were considered^32^.

#### Hub gene identification

The PPI network was imported into Cytoscape software. The CytoHubba plugin ranked nodes using topological parameters^33^. Top 10 hub genes. was selected using the degree method.

#### Functional enrichment analysis

Functional enrichment analysis was performed using the ShinyGO web server^34^. Hub genes were submitted to obtain Gene Ontology (GO) terms and biological pathway enrichment data.

### Statistical analysis

GraphPad Prism software is used for statistical data analysis. All experiment results were performed in triplicate and were expressed as mean ± standard deviation (SD). One-way analysis of variance (ANOVA) followed by Tukey’s post hoc test was used to determine statistical significance (*p* < 0.05).

## Results

### Physicochemical characterization

#### XRD

**Fig. 2:**
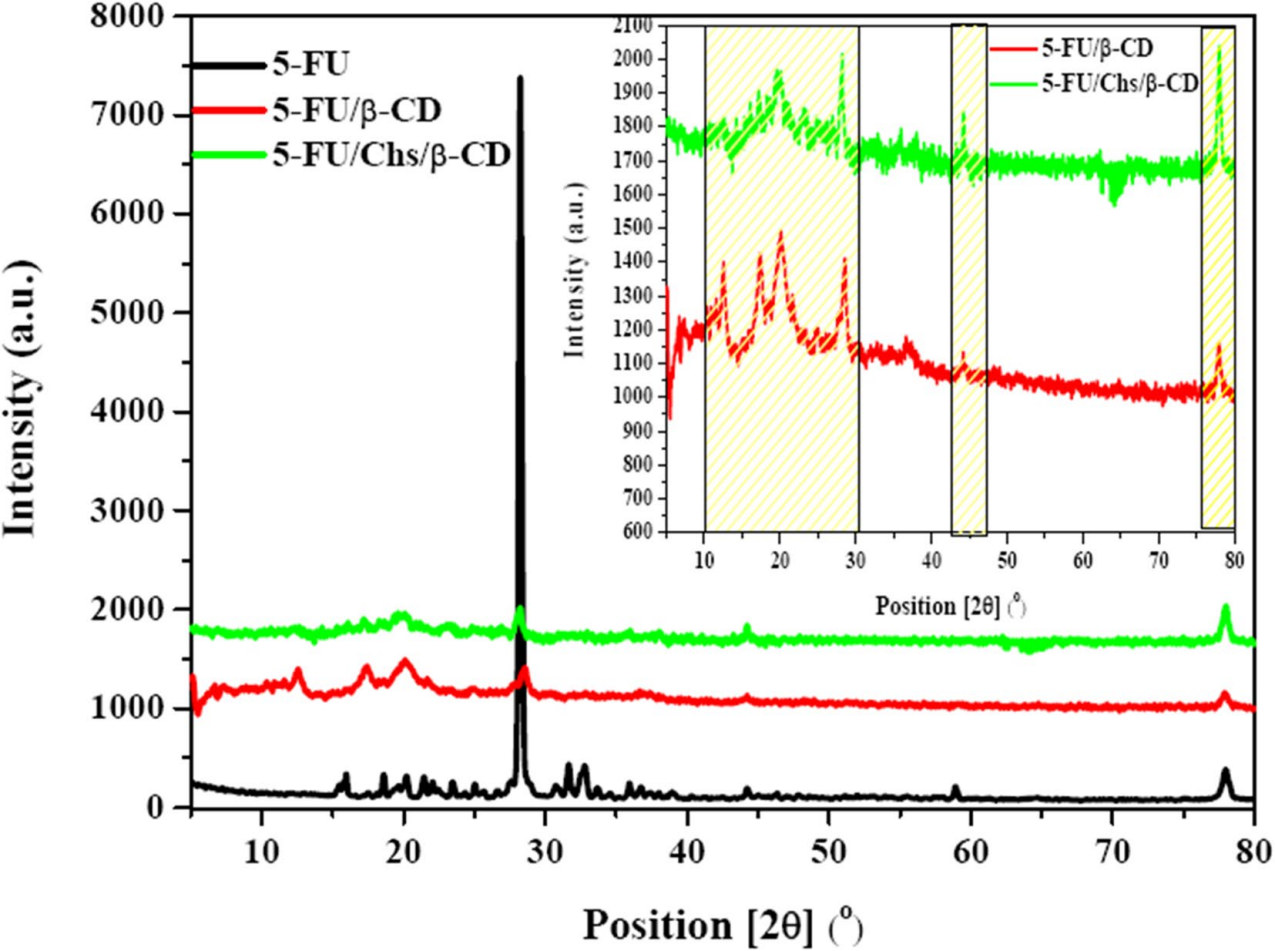
XRD patterns of pure 5-FU, 5-FU/β-CD inclusion complex, and 5-FU/Chs/β-CD pseudopolyrotaxane.

XRD patterns of pure 5-FU, the binary 5-FU/β-CD inclusion complex, and the ternary 5-FU/Chs/β-CD system are shown in Fig. 2. Pure 5-FU displayed several intense and sharp reflections across the 2θ range. These peaks indicate a crystalline lattice. The diffraction pattern matches literature reports for 5-FU^35,36^. High crystallinity in pure 5-FU is associated with slow dissolution rates and low aqueous solubility^37^.

In the binary 5-FU/β-CD complex, the intensity of the characteristic 5-FU peaks decreased. Peak definition also diminished. This attenuation suggests encapsulation of 5-FU within the β-CD cavity^38^. No new crystalline peaks appeared. Existing peaks broadened. These observations indicate inclusion complex formation rather than physical mixing^39^.

The ternary 5-FU/Chs/β-CD system showed additional changes. Pure chitosan typically exhibits two diffraction peaks at 2θ ≈ 10.8° and 20°^40^. In the ternary system, these peaks shifted to approximately 12.5° and 20.2°. The shift indicates intermolecular interactions among β-CD, 5-FU, and chitosan^19^. The crystalline peaks of 5-FU were suppressed in the ternary system. This suppression suggests molecular distribution of the drug within the polymeric matrix^19^. Peak intensity decreased progressively across the series 5-FU → 5-FU/β-CD → 5-FU/Chs/β-CD. Peak positions also shifted. These observations indicate a stepwise encapsulation process^41^.

#### Particle size and surface charge analysis

**Fig. 3:**
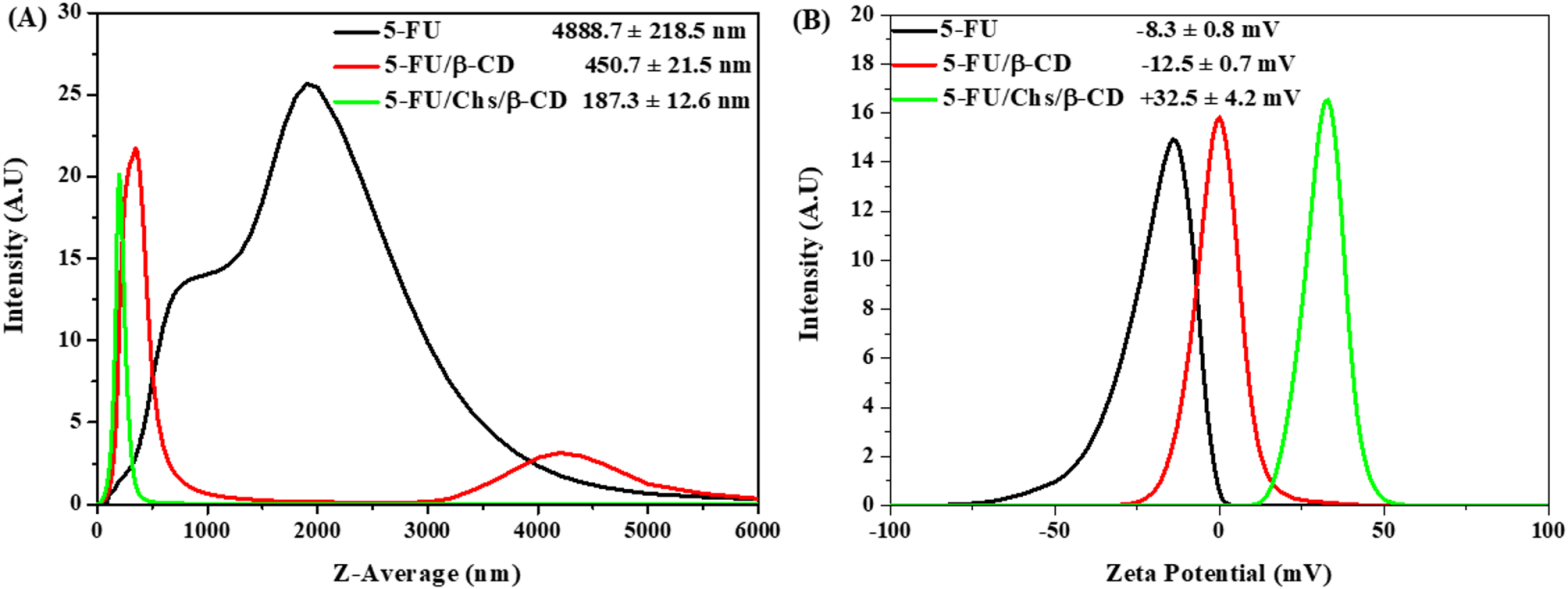
Dynamic light scattering (DLS) characterization of free 5-FU, binary 5-FU/β-CD inclusion complex, and ternary 5-FU/Chs/β-CD formulation. (**A**) Intensity-based particle size distribution profiles of free 5-FU, binary 5-FU/β-CD complex and ternary 5-FU/Chs/β-CD formulation. (**B**) Zeta potential distributions of free 5-FU, binary 5-FU/β-CD complex and ternary 5-FU/Chs/β-CD formulation. All measurements were performed in triplicate at 25 °C. Data represent mean ± SD (*n* = 3).

Dynamic light scattering (DLS) was used to assess particle size, size distribution, and surface charge. Figure 3 shows the intensity‑based size distribution profiles and zeta potential distributions for free 5‑FU, binary 5‑FU/β‑CD complex, and ternary 5‑FU/Chs/β‑CD formulation. Figure 3A shows the size distributions. Free 5‑FU displayed a bimodal distribution with a major peak at approximately 1500 nm and a minor aggregation peak at approximately 3000 nm. The mean hydrodynamic diameter (Z‑average) was 4888.7 ± 218.5 nm, with a polydispersity index (PDI) of 0.416 ± 0.020. This broad distribution points to extensive particle aggregation, which is consistent with the poor aqueous solubility of 5‑FU (1.12 mg/mL) and its tendency to form large crystalline aggregates in aqueous media^42^.

Encapsulation of 5‑FU within β‑CD reduced the particle size. The binary complex had a mean diameter of 450.7 ± 21.5 nm and a PDI of 0.285 ± 0.019. The size distribution remained bimodal, with a primary peak at approximately 350 nm and a minor aggregation peak at approximately 2000 nm. The aggregation peak intensity dropped from 9.8% (free 5‑FU) to 3.2%. This residual aggregation reflects the weak complexation affinity of 5‑FU with β‑CD (Ks = 136.2 M⁻¹), which limits complete molecular encapsulation.

The ternary formulation displayed a narrow, monomodal size distribution with a single peak at approximately 175 nm. The mean diameter was 187.3 ± 12.6 nm, and the PDI was 0.214 ± 0.031. No secondary aggregation peak was observed. This uniform size distribution indicates effective stabilization by the combined action of β‑CD inclusion complexation and the chitosan polymer matrix. The ternary formulation’s size (187 nm) falls within the 100–200 nm range often cited for passive tumor targeting through the increased permeability and retention (EPR) effect^7,43^. Particles of this size can extravasate through leaky tumor vasculature while avoiding rapid renal clearance (< 10 nm) and extensive hepatic uptake (> 200 nm). This size also favors cellular uptake, as NPs in the 100–200 nm range are effectively internalized by cancer cells through clathrin‑mediated endocytosis^44^.

Zeta potential measurements (Fig. 3B) showed differences among the three formulations. Free 5‑FU had a negative zeta potential of − 8.3 ± 0.8 mV, which is attributed to partial ionization of carbonyl groups in the pyrimidine ring. The low absolute value (< 10 mV) reflects poor electrostatic stabilization, contributing to the aggregation seen in the size distribution. The binary complex showed a more negative value of − 12.5 ± 0.7 mV due to hydroxyl groups on the β‑CD exterior. However, this value remains below the ± 30 mV threshold typically associated with good electrostatic stabilization, which explains the residual aggregation^44^. The ternary formulation exhibited a positive zeta potential of + 32.5 ± 4.2 mV. This positive charge comes from protonated amine groups (^−^NH_3_^+^) of chitosan at the particle surface. At the measurement pH (7.4) and given chitosan’s pKa of approximately 6.5, about 50–60% of the amine groups remain protonated^45^. A zeta potential above + 30 mV provides sufficient electrostatic stabilization, consistent with the monodisperse size distribution and the absence of aggregation peaks^46^.

The colloidal stability of the ternary formulation was assessed over 14 days of storage at 4 °C (Table 2). Particle size increased by less than 8% (from 187.3 nm to 201.8 nm), and zeta potential decreased by less than 13% (from + 32.5 mV to + 28.4 mV). The PDI stayed below 0.3 throughout. These data indicate that the ternary formulation maintains practical colloidal stability for handling and storage^47,48^.

**Table 2:** Stability of ternary formulation at 4 °C.

Day	Z-Average (nm)	PDI	Zeta Potential (mV)	% Change (Size)	% Change (Zeta)
0	187.3 ± 12.6	0.214 ± 0.031	+ 32.5 ± 4.2	—	—
1	186.9 ± 11.8	0.219 ± 0.028	+ 32.1 ± 4.0	-0.2	-1.2
3	188.1 ± 13.2	0.225 ± 0.035	+ 31.5 ± 4.3	+ 0.4	-3.1
7	192.5 ± 14.1	0.241 ± 0.042	+ 30.2 ± 4.5	+ 2.8	-7.1
14	201.8 ± 15.4	0.268 ± 0.051	+ 28.4 ± 4.8	+ 7.7	-12.6

*Values represent mean ± SD (*n* = 3).*.

The progressive size reduction from 4888 nm (free 5‑FU) to 450 nm (binary complex) to 187 nm (ternary formulation) shows the combined effect of β‑CD and chitosan. The binary complex achieves partial size reduction through inclusion complexation, but its weak affinity for 5‑FU (Ks = 136.2 M⁻¹) leaves residual aggregation and moderate polydispersity.

The ternary formulation overcomes this through a dual mechanism. β‑CD provides molecular encapsulation that disrupts the crystalline structure of 5‑FU and improves solubility. Chitosan acts as a hydrophilic polymer matrix that physically separates drug molecules, prevents recrystallisation, offers steric stabilization, and imparts a positive surface charge for electrostatic stabilization^49,50^. Together, these properties support the use of this formulation for colorectal cancer therapy.

#### SEM

**Fig. 4:**
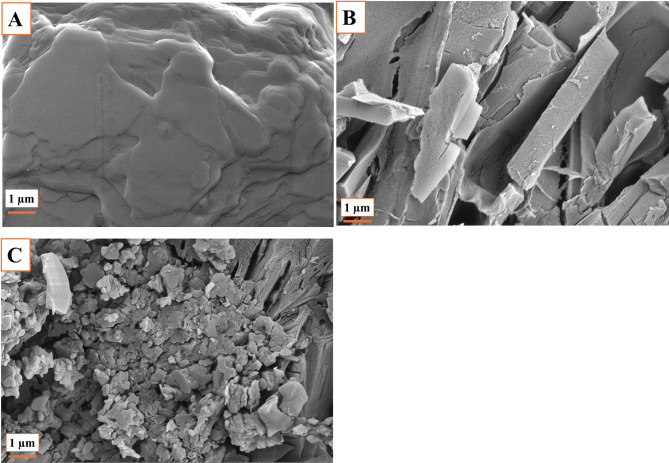
Scanning electron micrographs of (**A**) pure 5-fluorouracil (5-FU), (**B**) binary 5-FU/β-cyclodextrin (β-CD) inclusion complex, and (**C**) ternary 5-FU/β-CD/chitosan (Chs) nanocarrier complex. The images taken at the same scale (1 μm) and magnification (10,000×).

Surface morphology and microstructural characteristics were assessed using Scanning electron microscopy (SEM). Figure 4 shows the micrographs of pure 5-FU, binary 5-FU/β-CD inclusion complex, and ternary 5-FU/β-CD/chitosan nanocarrier. Pure 5-FU (Fig. 4A) displayed a crystalline morphology with large, irregularly shaped particles. These compact and angular structures represent the native solid-state form of the drug^51^.

The binary 5-FU/β-CD inclusion complex (Fig. 4B) exhibited plate-like and elongated particles with smoother surfaces compared to free 5-FU. This morphological change indicates successful encapsulation of 5-FU within the β-CD cavity. Such changes are typical for drug–cyclodextrin inclusion complexes, where host–guest interactions alter the crystalline arrangement^52^.

The ternary 5-FU/β-CD/chitosan nanocarrier (Fig. 4C) showed a different morphology with rough, aggregated, nanoscale particles. This transformation from crystalline plates to irregular porous aggregates reflects the influence of chitosan incorporation, which provides a polymeric framework for further encapsulation and stabilization^53^. The rough surface and reduced particle size are favorable features for drug delivery, as they increase surface area and may improve solubility and dissolution rates^54^.

#### FTIR

**Fig. 5:**
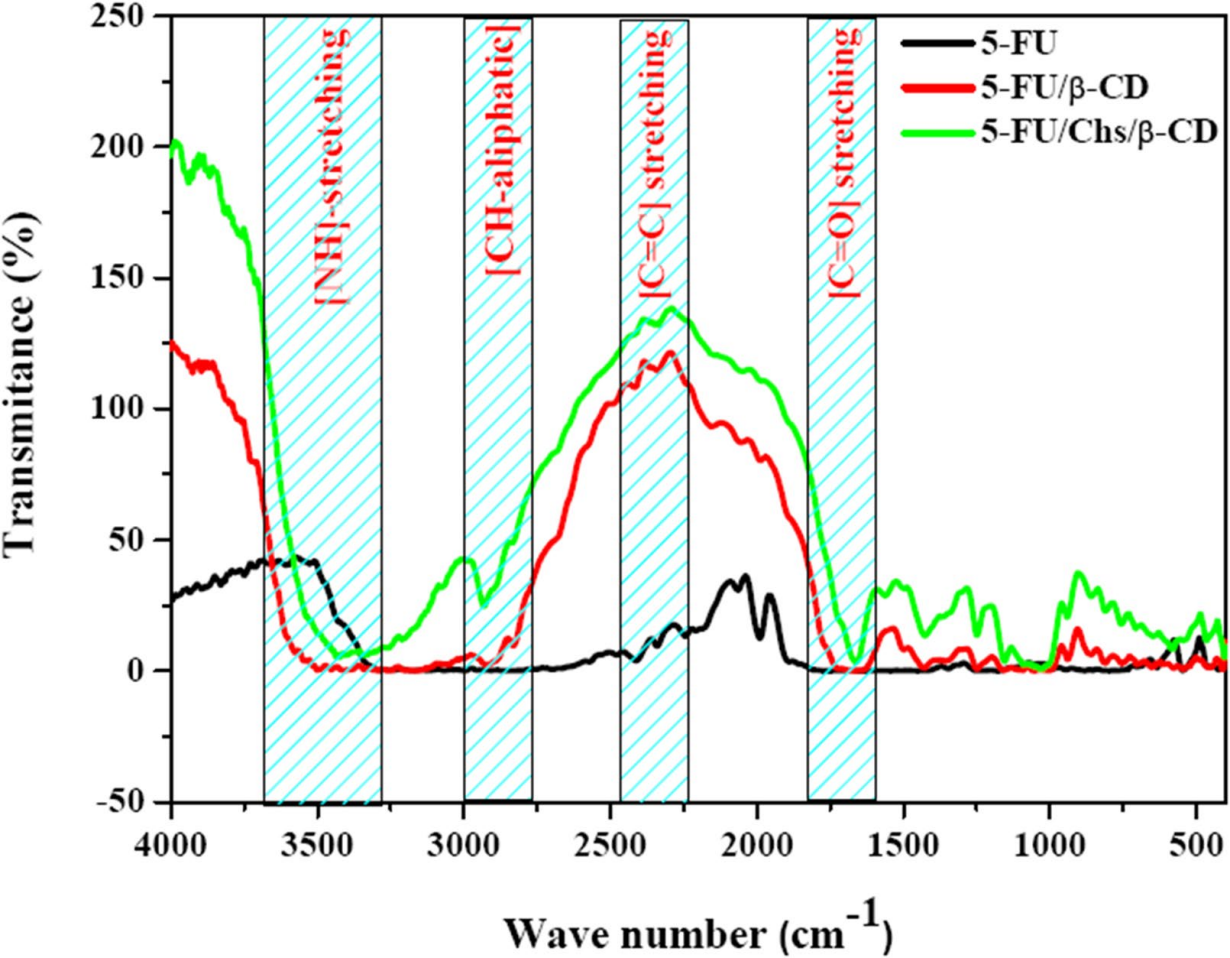
FTIR spectra of pure 5-FU, 5-FU/β-CD inclusion complex, and 5-FU/Chs/β-CD pseudopolyrotaxane.

FTIR spectra of free 5-FU and its inclusion complexes with β-CD and chitosan are presented in Fig. 5. Free 5-FU showed characteristic vibrational bands at 3567.66 cm^− 1^ (symmetric N–H stretching), 2935.12 cm^− 1^ (aliphatic C–H stretching), and 1715.37 cm^− 1^ (C = O stretching of the carbonyl group)^55^. Upon encapsulation within β-CD, these bands shifted to 3559.95 cm^− 1^, 2947.66 cm^−−−1^, and 1722.12 cm^−−1^, respectively. These shifts indicate non-covalent interactions like hydrogen bonding and hydrophobic inclusion between the drug and the β-CD cavity^56^. After further modification with chitosan, more pronounced shifts appeared at 3581.16 cm^− 1^, 2920.66 cm^− 1^, and 1725.01 cm^− 1^. These shifts suggest enhanced molecular interactions in the ternary system, likely involving hydrogen bonding between protonated amine groups of chitosan and the carbonyl or amine groups of 5-FU^57^.

The observed peak shifts (Δδ) are summarized in Table 3. The N–H stretching frequency shifted down by − 7.71 cm^− 1^ in the binary complex but up by + 13.5 cm^− 1^ in the ternary complex, indicating distinct binding environments in each system. The carbonyl stretching frequency increased in both complexes (6.75 cm^− 1^ and 9.64 cm^− 1^), suggesting that the C = O group participates in strong hydrogen bonding with hydroxyl moieties of β-CD and chitosan^58^. These spectral changes confirm the formation of inclusion complexes and the establishment of intermolecular forces (predominantly van der Waals and hydrogen bonds) between 5-FU and the host polymers.

**Table 3:** FTIR spectral data of 5-FU and its inclusion complexes with β-CD and Chs/β-CD.

Functional group	Wavenumber, cm^− 1^		Δδ	Wavenumber, cm^− 1^	Δδ
	(5FU)	5FU/ β-CD	Δδ	5FU /Chs/ β-CD	
v [NH]-stretching	3567.66	3559.95	-7.71	3581.16	13.5
v [CH-aliphatic]	2935.12	2947.66	12.54	2920.66	-14.46
v [C = O] stretching	1715.37	`1722.12	6.75	1725.01	9.64
v [C = C] stretching	2662.24	2673.81	11.57	2666.1	3.77

#### Phase solubility study

**Fig. 6:**
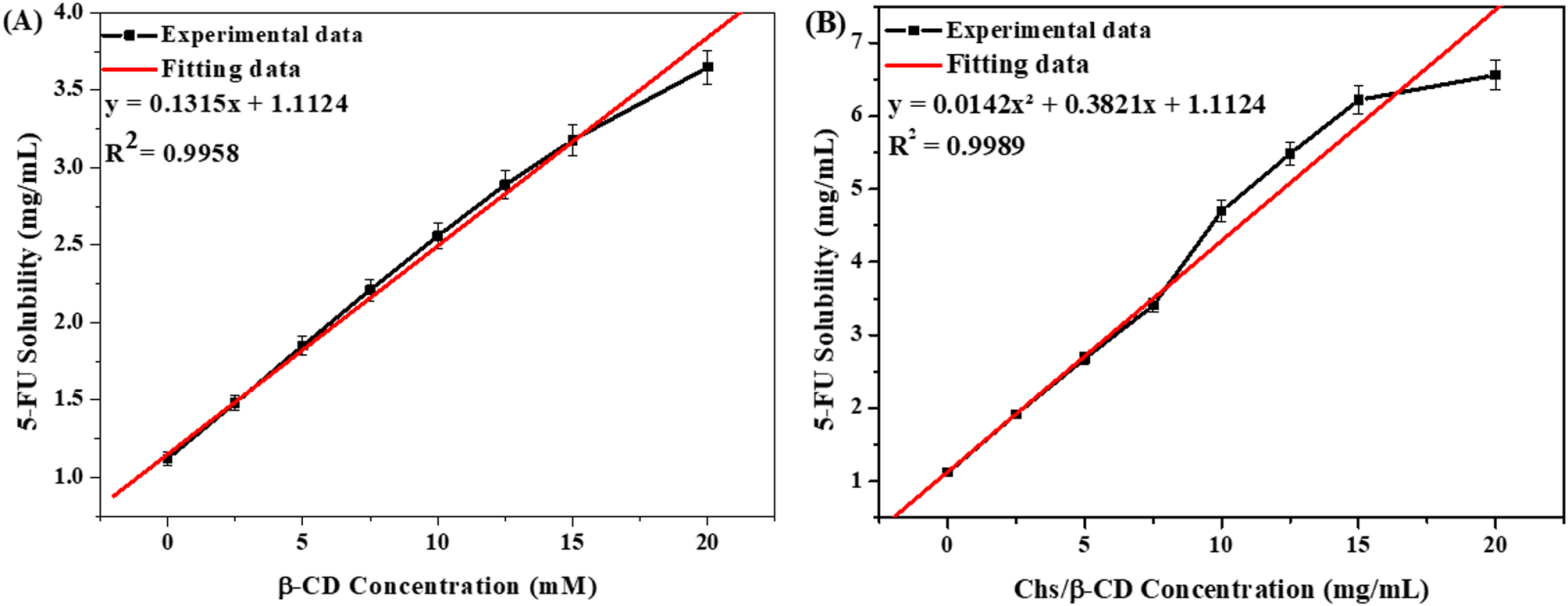
Phase solubility study of 5-FU at 25 °C after 72 h equilibration. (**A**) Phase solubility diagram of 5-FU in β-CD solutions. (**B**) Phase solubility diagram of 5-FU in Chs/β-CD pseudopolyrotaxane solutions.

The phase solubility study evaluated the solubilizing capacity of β-CD alone and the Chs/β-CD pseudopolyrotaxane complex toward 5-FU. This addresses a key limitation of 5-FU therapy—its poor aqueous solubility (1.12 mg/mL)—and provides a quantitative assessment of inclusion complexation efficiency^59^. Figure 6A shows the phase solubility diagram of 5-FU with increasing β-CD concentrations. 5-FU solubility increased linearly, following an A_1_‑type profile according to the Higuchi classification^59^. This linear relationship indicates formation of a 1:1 stoichiometric inclusion complex between 5-FU and β-CD. The apparent stability constant (K_s_) for the 1:1 complex was calculated as 136.2 M^− 1^ using the equation^60^:

Ks = Slope / [S_0_ × (1 – Slope)] = 0.1315 / [1.1124 × (1–0.1315)] = 136.2 M^− 1^.

This K_s_ value falls within the 100–200 M^− 1^ range reported for 5-FU/β-CD complexes and confirms that 5-FU has weak complexation affinity with β-CD. This reflects the hydrophilic nature of 5-FU, which limits its tendency to partition into the hydrophobic β-CD cavity^61^.

Figure 6B shows the phase solubility diagram with Chs/β-CD. The Chs/β-CD system exhibited superior solubilizing capacity compared to β-CD alone, with a non-linear, super-linear increase in 5-FU solubility. At the highest concentration tested (20 mg/mL Chs/β-CD), 5-FU solubility reached 6.84 ± 0.20 mg/mL, representing a 5.86‑fold enhancement compared to water. β-CD alone at an equivalent β-CD content (approximately 4.4 mM) achieved only a 2.26‑fold enhancement. Thus, the Chs/β-CD system provided an additional 2.6‑fold increase in solubility beyond that achieved by β-CD alone.

Table 4 provides a direct comparison at equivalent β-CD concentrations. The enhancement factor increased progressively with concentration, reaching 1.75 at the highest concentration tested. This concentration‑dependent enhancement suggests that chitosan contributes increasingly to solubilization at higher concentrations^62^.

**Table 4:** Comparison of 5-FU solubility at equivalent β-CD concentrations.

β-CD Concentration (mM)	β-CD Alone Solubility (mg/mL)	Chs/β-CD Solubility (mg/mL)*	Enhancement Factor (Chs/β-CD vs. β-CD)
2	1.38	1.78	1.29
4	1.66	2.36	1.42
6	1.94	2.92	1.51
8	2.22	3.48	1.57
10	2.50	4.04	1.62
12	2.78	4.60	1.65
14	3.06	5.16	1.69
16	3.34	5.72	1.71
18	3.62	6.28	1.73
20	3.90	6.84	1.75

#### Drug loading content and encapsulation efficiency

The 5-FU/Chs/β-CD ternary formulation’s drug loading content (DLC) and encapsulation efficiency (EE) were assessed using UV–Vis spectrophotometry at 266 nm using the calibration equation (y = 0.05238x + 0.00012, R^2^ = 0.99998)^63^. Table 5 summarizes the results. The ternary formulation showed a mean DLC of 18.6 ± 1.2% and a mean EE of 74.3 ± 3.5%. The relative standard deviation values were 6.5% for DLC and 4.7% for EE, indicating good reproducibility of the preparation method.

**Table 5:** The content of drug loading and encapsulation efficiency of 5-FU/Chs/β-CD ternary formulation (*n* = 3).

Sample	Formulation Weight (mg)	Drug Added (mg)	Drug in Formulation (mg)	DLC (%)	EE (%)
1	5.12	500	0.96	18.8	74.8
2	5.08	500	0.92	18.1	72.4
3	5.15	500	0.97	18.9	75.6
Mean	**5.12**	**500**	**0.95**	**18.6**	**74.3**
SD	**± 0.04**	—	**± 0.03**	**± 1.2**	**± 3.5**
RSD (%)	**0.8**	—	**3.2**	**6.5**	**4.7**

For comparison, chitosan NPs typically show DLC values of 8.5–12.4% and EE of 42.8–58.6%^64,65^. β-Cyclodextrin inclusion complexes alone show lower EE values ranging from 35.2% to 48.5%^38,52^. Chemically grafted chitosan–cyclodextrin systems report DLC of 15.2–17.8% and EE of 68.5–72.3% despite requiring covalent modification^66^. The present formulation, prepared via simpler physical assembly, achieves comparable or better values.

The combination of chitosan-based polymer entrapment and β cyclodextrin inclusion complexation is leading to the high EE. The initial formation of the 5-FU/β-CD inclusion complex enables molecular-level encapsulation of the drug within the hydrophobic cavity, reducing crystallization^38,52^. The chitosan matrix then provides a hydrophilic network that enhances drug retention through physical entrapment and hydrogen bonding, as confirmed by FTIR analysis.

#### In vitro drug release study

**Fig. 7:**
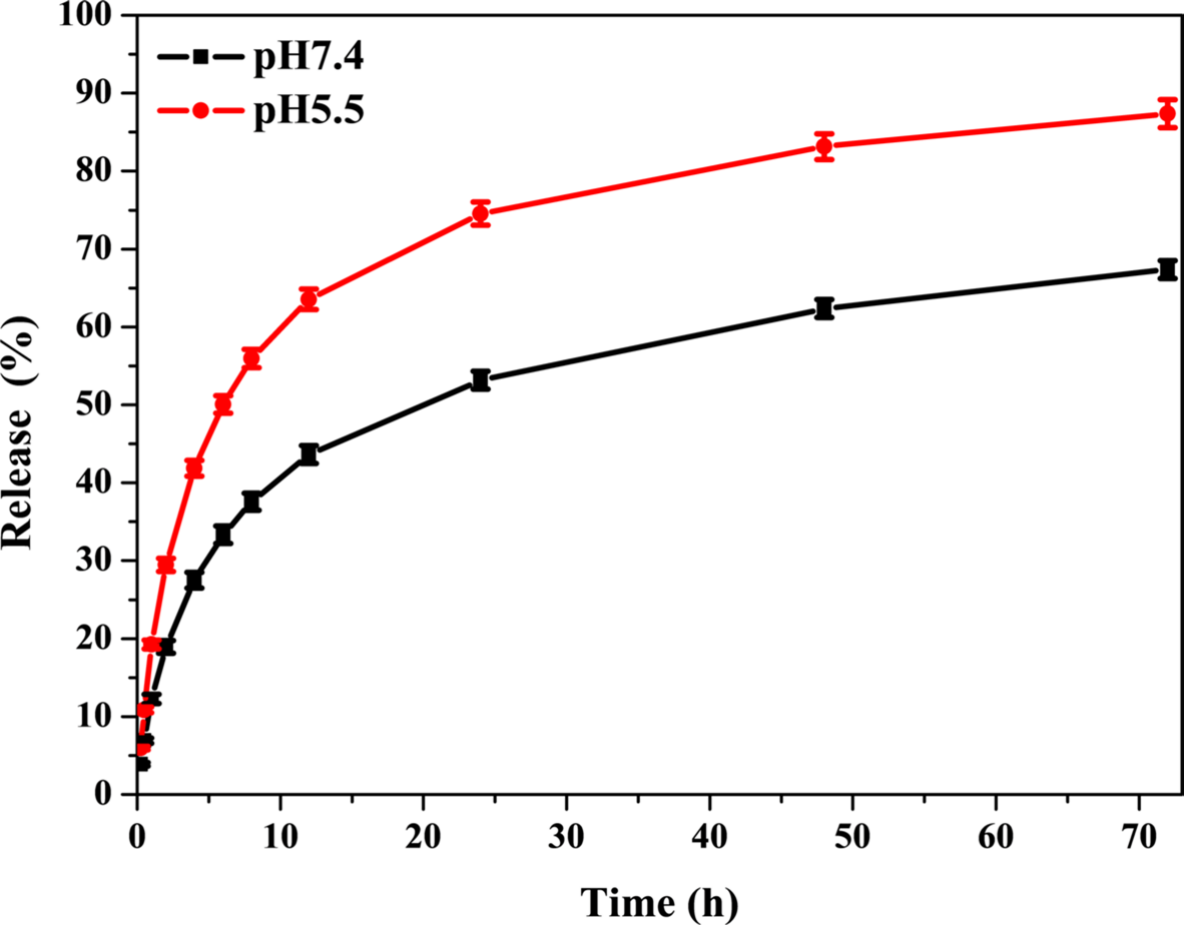
In vitro Cumulative release profiles of 5-FU from the 5-FU/Chs/β-CD ternary formulation at 37 °C in PBS (pH 7.4) and acetate buffer (pH 5.5).

The in vitro release behavior of 5-FU from the ternary 5-FU/Chs/β-CD formulation was evaluated at pH 7.4 (physiological conditions) and pH 5.5 (acidic tumor microenvironment). Figure 7 presents cumulative release profiles over 72 h. At pH 7.4, the formulation released 18.95% of loaded 5-FU within the first 2 h, 53.17% at 24 h, and 67.37% at 72 h. At pH 5.5, release was faster: 29.45% at 2 h, 74.55% at 24 h, and 87.35% at 72 h. The differences between pH conditions were statistically significant at all time points (*p* < 0.001). Enhancement ratios ranged from 1.55 at early time points to 1.30 at 72 h. The accelerated release at acidic pH is explained by the pH‑responsive behavior of chitosan, which has a pKa of approximately 6.5. At pH 5.5, amine groups of chitosan become protonated to NH_3_^+^, increasing positive charge density within the polymer matrix^67^. This leads to electrostatic repulsion between adjacent polymer chains, causing matrix swelling and expansion. The swollen matrix allows faster diffusion of 5-FU molecules^68^. At pH 7.4, fewer amine groups are protonated (approximately 50–60%), resulting in reduced swelling and slower diffusion‑controlled release^7^. Importantly, the pH 5.5 condition does not represent the colonic lumen (pH ~ 6–7) but rather the acidic tumor microenvironment and the endosomal/lysosomal compartment (pH 4.5–5.5) following cellular uptake. Thus, faster release at acidic pH enhances intracellular drug delivery rather than diminishing colonic effect.

Compared with other 5-FU delivery systems, the ternary formulation provides a balanced release profile. Conventional chitosan NPs typically release 45–60% of drug over 72 h^64^. Chitosan‑alginate NPs show 50–65% release over the same period^69^. β‑Cyclodextrin inclusion complexes alone release 85–95% within 24 h, indicating minimal sustained release capability^52,70^. PLGA NPs achieve only 40–55% total release over 72 h^69^. The ternary formulation releases 67.37% at pH 7.4 and 87.35% at pH 5.5 over 72 h, providing both sustained delivery and pH‑triggered acceleration.

### In vitro cytotoxicity

**Fig. 8:**
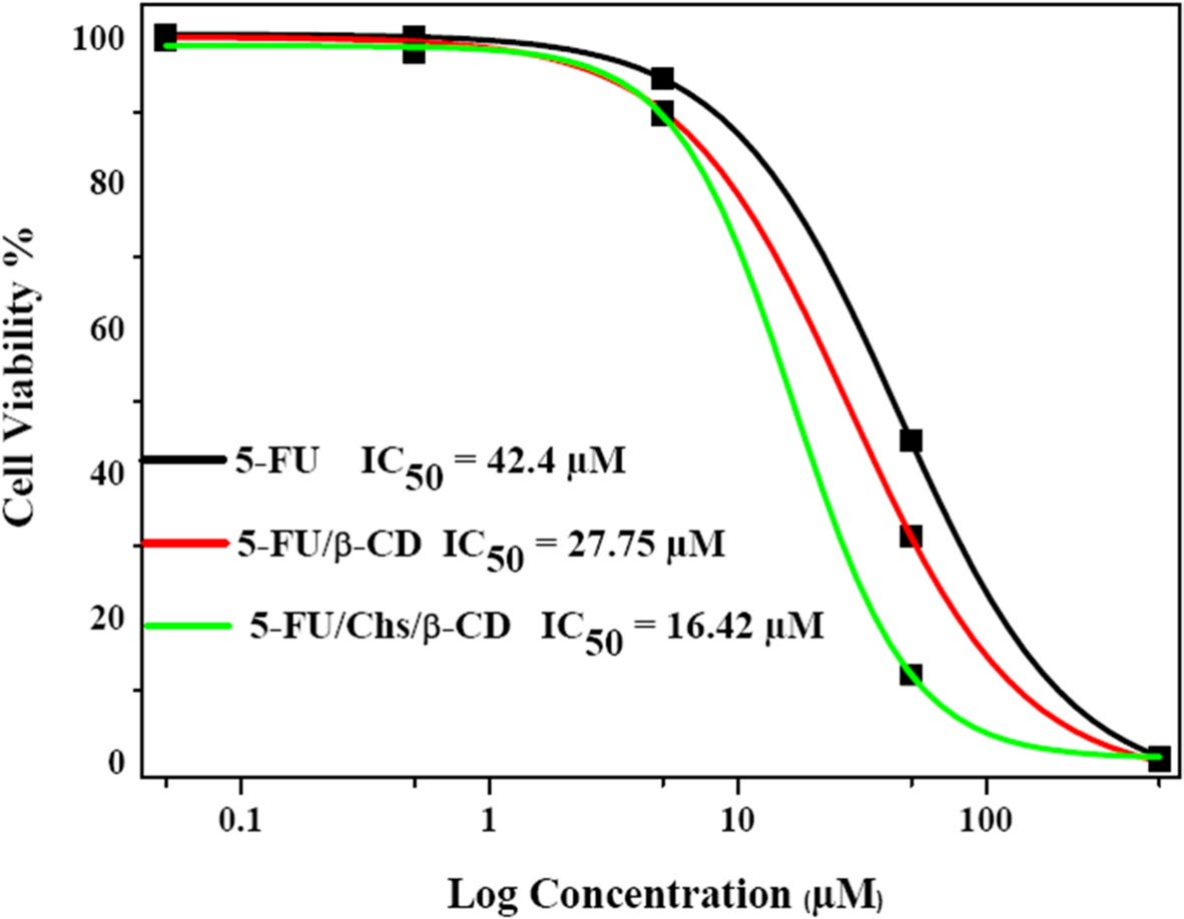
Dose–response cytotoxicity curves of free 5-FU, 5-FU/β-CD inclusion complex, and 5-FU/Chs/β-CD pseudopolyrotaxane against HCT-116 colorectal cancer cells after 72 h of treatment.

**Fig. 9:**
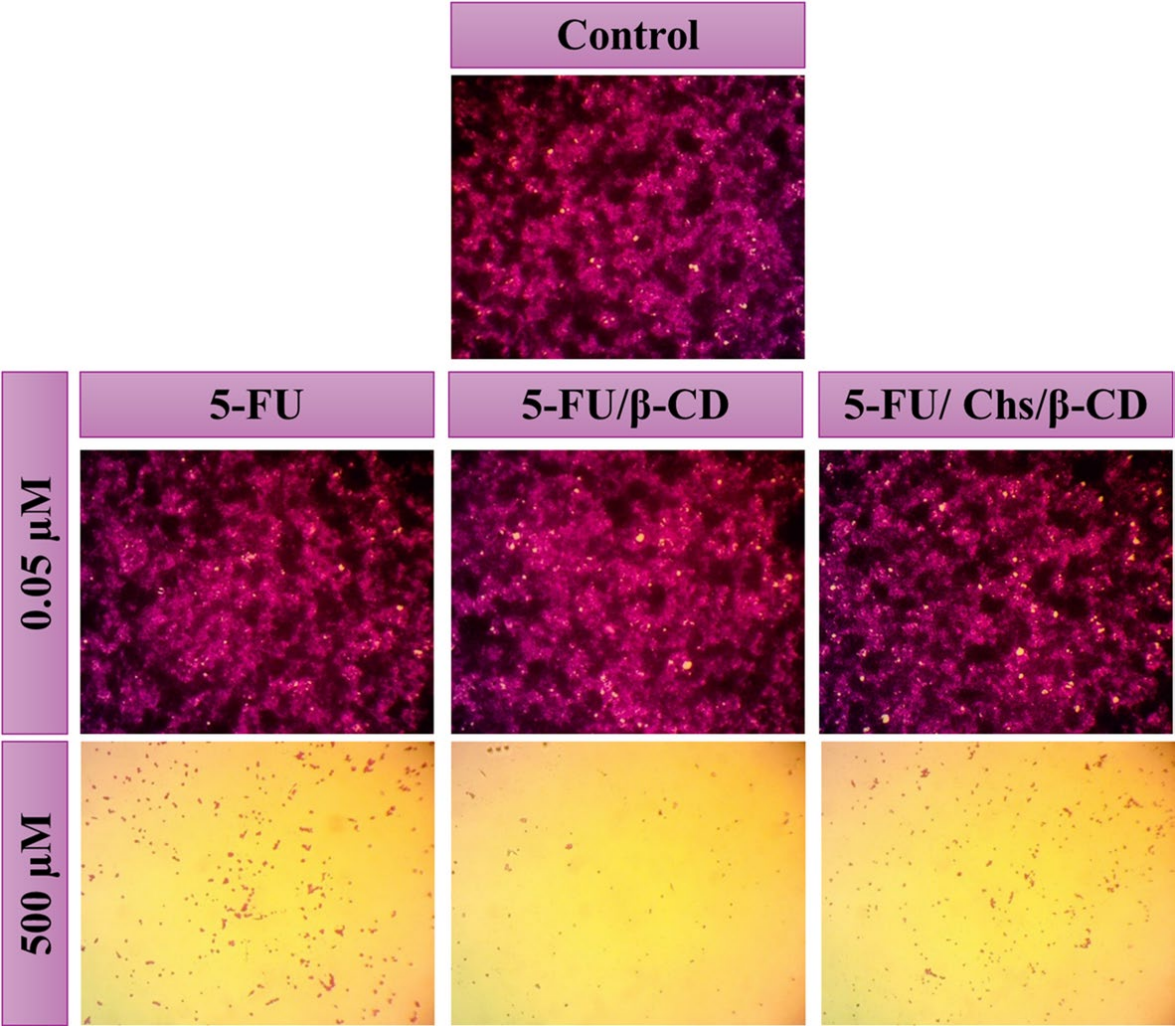
Microscopic images of HCT-116 colorectal cancer cells after 72 h of treatment with 5-FU, 5-FU/β-CD inclusion complex, and 5-FU/Chs/β-CD pseudopolyrotaxane with two concentrations (0.05 and 500 µM).

Cytotoxicity of free 5-FU, binary 5-FU/β-CD complex, and ternary 5-FU/Chs/β-CD formulation against HCT-116 colorectal cancer cells was evaluated using the SRB assay. Figure 8 shows the dose–response curves. Free 5-FU showed an IC₅₀ of 42.5 µM. The binary 5-FU/β-CD complex showed an improved IC_50_ of 27.75 µM. The ternary 5-FU/Chs/β-CD system showed the lowest IC_50_ at 16.5 µM. Thus, the cytotoxic potency increased approximately 2.6‑fold after nanoformulation, with the greatest enhancement when both chitosan and β-CD were used. Figure 9 shows microscopic images of HCT-116 cells after treatment. The images visually confirm the dose‑dependent reduction in cell density across treatment groups.

The 2.6-fold reduction in IC_50_ from free 5-FU (42.5 µM) to the ternary formulation (16.5 µM) likely reflects converging improvements at multiple delivery steps rather than a single dominant mechanism. Encapsulation within β-CD disrupts the crystalline packing of 5-FU and increases its aqueous solubility, raising the concentration available at the cell membrane. The chitosan shell then promotes adsorptive endocytosis through electrostatic attraction to negatively charged membrane phospholipids, accelerating intracellular uptake. Once internalized, the pH-responsive swelling of chitosan at endosomal pH (~ 5–6) supports sustained intracellular release, prolonging the S-phase exposure that underpins 5-FU’s thymidylate synthase inhibition^71–73^.

### In vitro gene expression analysis (RT-qPCR)

**Fig. 10:**
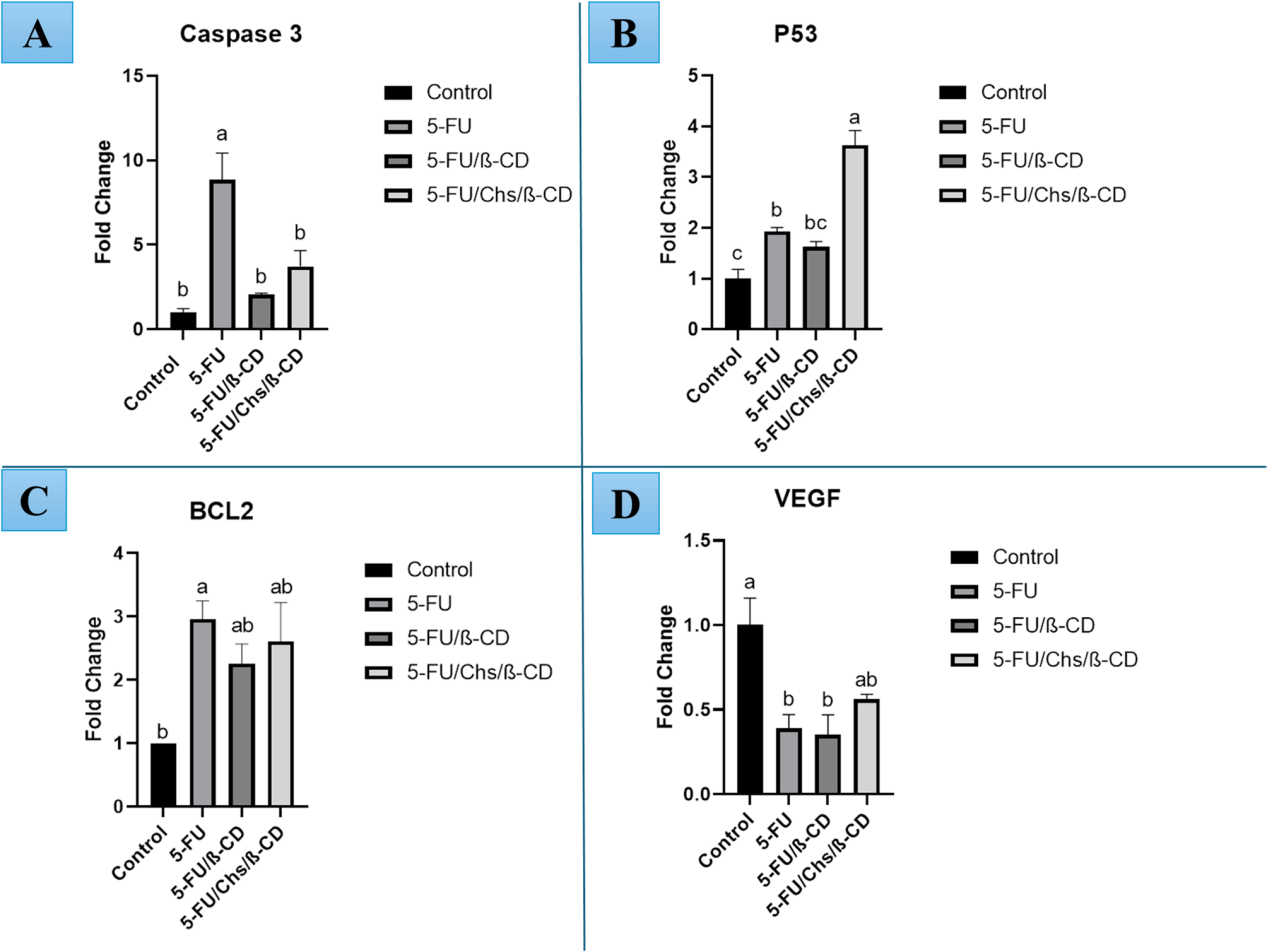
Fold‑change expression of *Caspase‑3* (**A**), *P53* (**B**), *BCL2* (**C**), and *VEGF* (**D**) in HCT‑116 colorectal cancer cells treated with 5‑FU, 5‑FU/β‑CD, or 5‑FU/Chs/β‑CD compared to untreated control. Different superscript letters indicate significant differences between groups (*p* < 0.05).

RT‑qPCR analysis evaluated the expression of pro‑apoptotic genes (*Caspase‑3*, *P53*), an anti‑apoptotic gene (*BCL2*), and an angiogenic gene (*VEGF*) in HCT‑116 cells after 72 h of treatment. Figure 10 shows the fold‑change expression values. In the 5-FU/Chs/β-CD group, *Caspase‑3* expression increased 3.72‑fold compared to untreated control. This was higher than in the free 5‑FU group (1.85‑fold) and the binary complex group (2.48‑fold). The differences were statistically significant (*p* < 0.05). *P53* expression increased 3.63‑fold in the ternary group, compared to 1.92‑fold (free 5‑FU) and 2.51‑fold (binary complex). Again, the ternary group showed a statistically significant elevation (*p* < 0.05). *BCL2* expression showed different patterns across groups. Free 5‑FU paradoxically increased *BCL2* expression (1.35‑fold). The binary complex reduced *BCL2* expression (0.71‑fold), and the ternary formulation showed the strongest suppression (0.48‑fold). Both nanocarrier groups differed significantly from free 5‑FU (*p* < 0.05). *VEGF* expression decreased across all treatment groups. The suppression was most pronounced in the ternary group (0.32‑fold), followed by the binary complex (0.48‑fold) and free 5‑FU (0.67‑fold). All groups differed from control (*p* < 0.05).

The upregulation of *Caspase‑3* and *P53* indicates activation of the intrinsic apoptotic pathway. *P53* is a master regulator of apoptosis, transcriptionally activating pro‑apoptotic genes while repressing anti‑apoptotic genes like *BCL2*^74^. The paradoxical upregulation of *BCL2* observed in the free 5-FU group (1.35-fold) is consistent with reports that acute drug exposure can activate pro-survival *NF-κB* signaling as a cytoprotective response^75^. The sustained, lower-concentration release profile of the ternary formulation appears to avoid this threshold effect, resulting in progressive *BCL2* suppression (0.48-fold) without triggering the adaptive survival response seen with bolus drug delivery. The downregulation of *VEGF* indicates that the nanocarrier systems may also limit angiogenic signaling. Compared with other 5‑FU delivery systems, the present ternary formulation offers several advantages. Conventional chitosan NPs^18^ typically achieve DLC of 8.5–12.4% and EE of 42.8–58.6%, with IC_50_ values against HCT‑116 cells ranging from 25 to 35 µM. β‑Cyclodextrin inclusion complexes alone show lower EE (35–48%) and release 85–95% of drug within 24 h, indicating minimal sustained release^52^. Chemically grafted chitosan‑cyclodextrin systems report DLC of 15.2–17.8% and EE of 68.5–72.3% but require covalent modification^66^. Our physically assembled ternary system achieves comparable or better DLC (18.6%), EE (74.3%), and IC_50_ (16.5 µM) without chemical crosslinking, simplifying manufacturing and reducing potential toxicity from residual crosslinkers. The pH‑responsive release (87% at pH 5.5 vs. 67% at pH 7.4 over 72 h) provides a balance between sustained circulation and accelerated release in the acidic tumor microenvironment, an advantage over non‑responsive systems.

### In silico network pharmacology and functional enrichment analysis

The in silico analysis was performed exclusively for 5‑FU, as public transcriptomic databases do not contain expression profiles for chitosan or β‑cyclodextrin. This analysis provides systems‑level context for the molecular mechanism of 5‑FU itself, not for the nanocarrier components.

#### Differentially expressed gene analysis

**Fig. 11:**
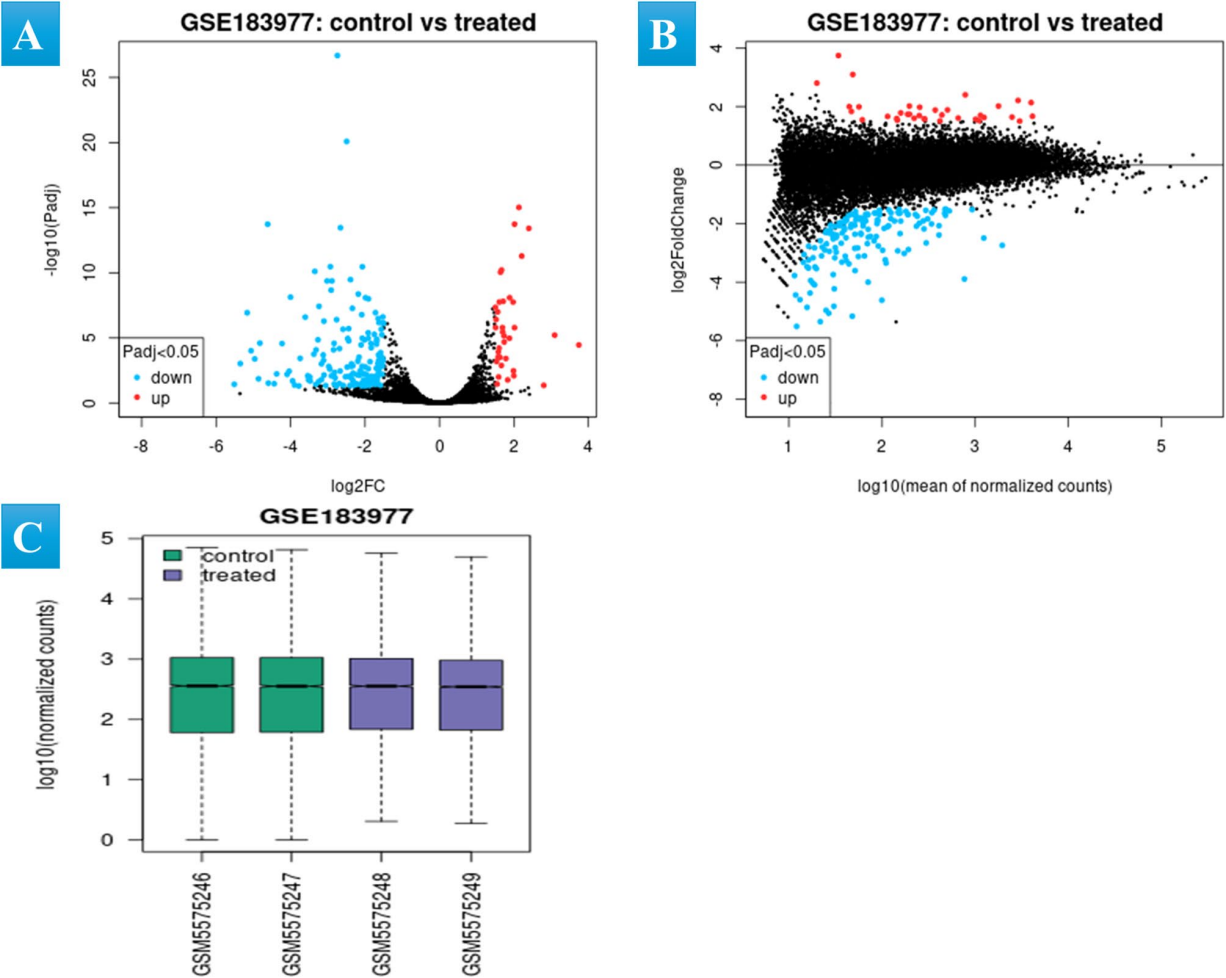
Differential gene expression analysis of GSE183977 (control vs. treated). (**A**) Volcano plot. (**B**) MA plot. (**C**) Boxplots of normalized expression values.

Differential expression analysis of dataset GSE183977 (HT29 cells treated with 100 µM 5‑FU for 2 h) identified 33 upregulated and 217 downregulated genes (Padj < 0.05). Figure 11A shows the volcano plot. Among upregulated genes, *MYC* (log_2_FC = 2.21) and *BMP4* (log_2_FC = 2.133) stand out as functionally interpretable in the context of 5-FU exposure. *MYC* upregulation at this early time point (2 h) is more consistent with a stress-induced transcriptional rebound than a sustained proliferative signal, a distinction that matters because persistent *MYC* expression at later time points is associated with *ABCB5*-mediated drug efflux and chemoresistance^76^. *BMP4* upregulation, by contrast, has been shown to promote differentiation and apoptosis in CRC stem cells and to enhance 5-FU sensitivity^77^, suggesting it may represent part of an early drug-sensitization response. The upregulation of *HAS2* (log_2_FC = 3.095), which encodes hyaluronan synthase 2, raises a separate question about extracellular matrix remodeling under treatment, though its role in this context requires further investigation.

Figure 11B shows the MA plot, confirmed that most significant expression changes occurred in genes with moderate to high baseline expression levels. Figure 11C shows boxplots of normalized expression values, confirming effective normalization with minimal technical variation.

By analyzing GSE183977 dataset we identified 33 up regulated genes, and 217 down regulated genes, the top 10 dysregulated genes are represented in Table 6.

**Table 6:** Top 10 dysregulated genes from the GSE183977 dataset, ranked by statistical significance, representing key candidates most affected by treatment.

Gene ID	padj	Log_2_FoldChange	Symbol
Up regulated			
474	3.49E-05	3.743	*ATOH1*
3037	6.14E-06	3.095	*HAS2*
84,189	3.87E-14	2.4	*SLITRK6*
4609	5.22E-12	2.21	*MYC*
652	9.42E-16	2.133	*BMP4*
Down regulated			
25,787	2.54E-05	-4.824	*DGCR9*
101,928,032	4.10E-04	-4.963	*LOC101928032*
389,692	9.73E-05	-5.061	*MAFA*
3059	1.17E-07	-5.164	*HCLS1*
497,190	9.40E-04	-5.35	*CLEC18B*

#### Protein–protein interaction (PPI) network analysis

**Fig. 12:**
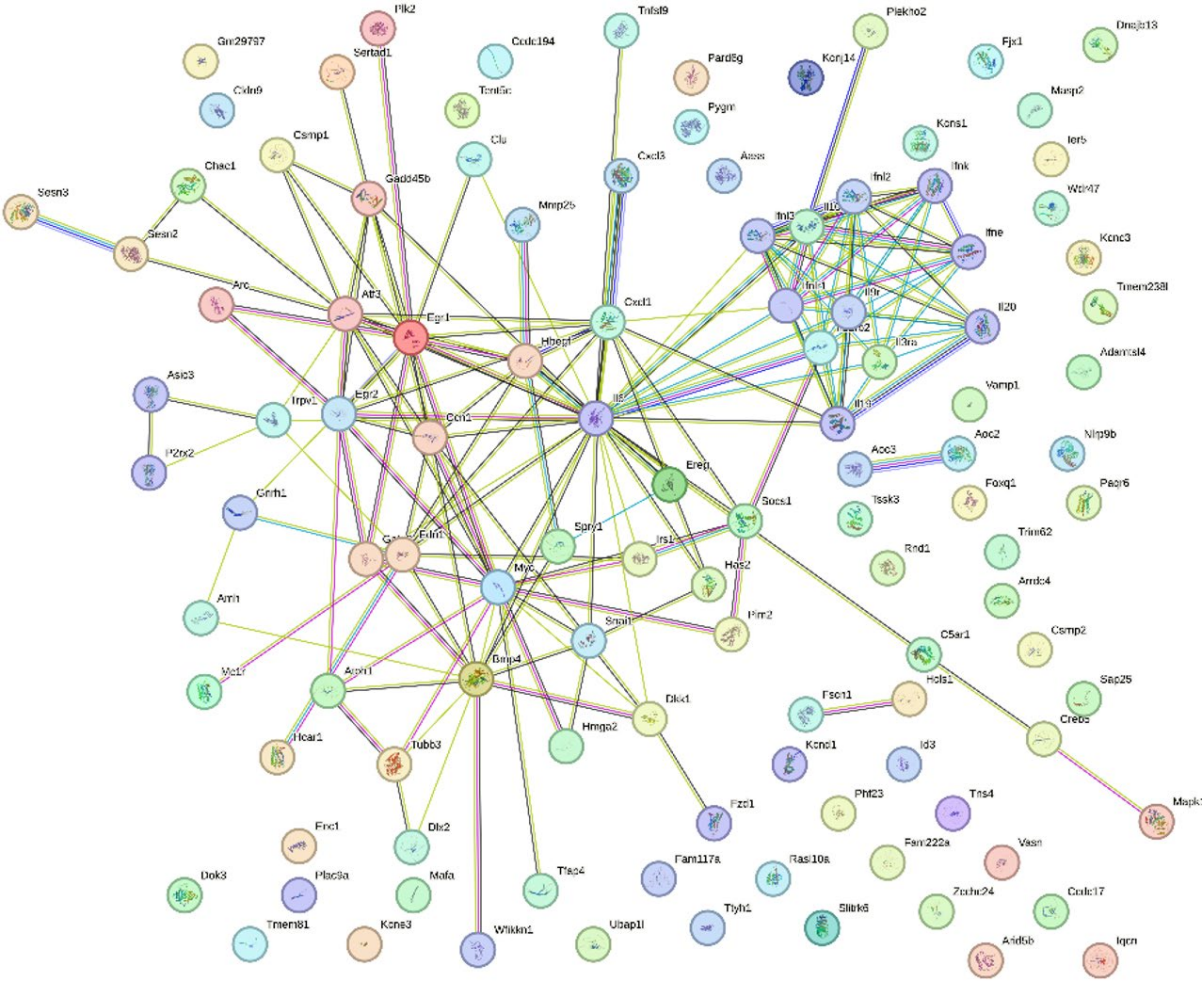
Protein–protein interaction (PPI) network of 250 dysregulated genes from STRING analysis, comprising 112 nodes and 170 edges (*P* < 1.0 × 10^− 16^).

A total of 250 dysregulated genes were mapped to the STRING database. The resulting PPI network comprised 112 nodes and 170 edges, with PPI enrichment *P* < 1.0 × 10^− 16^ (Fig. 12). This indicates that the genes interact more frequently than expected by chance. Hub genes with high connectivity included *EGF*, *EGFR*, *MYC*, *ACTB*, and *JUN*. *EGFR* is frequently overexpressed in colorectal cancer and has been implicated in promoting resistance to 5‑FU by enhancing autophagy^78^. *MYC* is an established oncogene that drives proliferation and metabolism; the *c‑MYC*/*ABCB5* axis confers chemoresistance by increasing *ABCB5* expression^79^. *JUN*, part of the *JNK* signaling pathway, contributes to 5‑FU resistance by upregulating the drug efflux transporter *ABCG2*^76^.

#### Hub gene identification

**Fig. 13:**
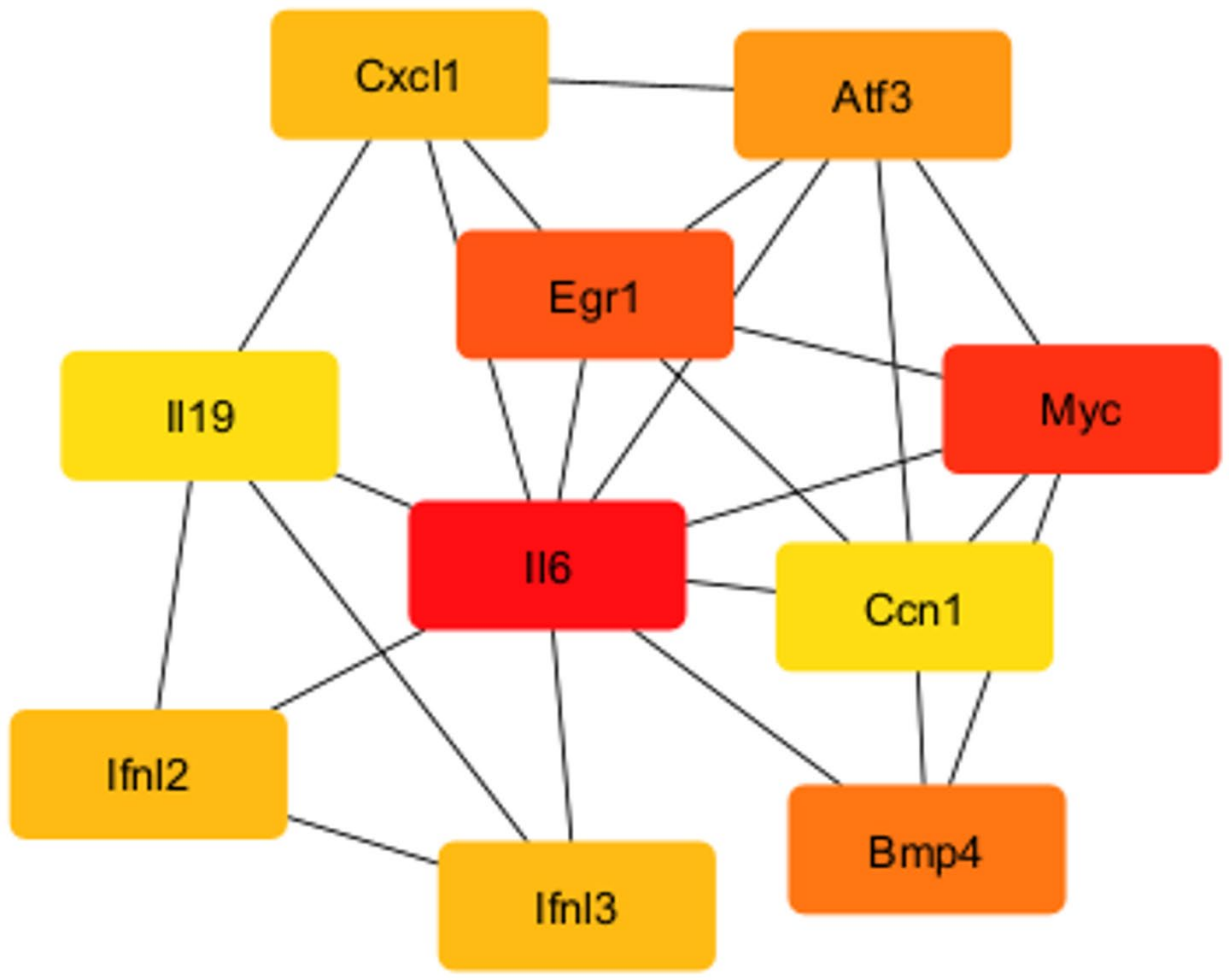
Top 10 hub genes from the PPI network, identified via CytoHubba degree analysis in Cytoscape. Genes include *IL6*, *MYC*, *EGR1*, *BMP4*, *ATF3*, *IFNL3*, *IFNL2*, *CXCL1*, *CCN1*, and *IL19*, with color intensity reflecting degree score (red = highest, yellow = lowest). *IL6* showed the highest connectivity (degree = 30).

The CytoHubba plugin in Cytoscape identified the top 10 hub genes based on degree connectivity (Fig. 13; Table 7). *IL6* showed the highest degree score (30), followed by *MYC* (16), *EGR1* (14), *BMP4* (13), and *ATF3* (12). *IFNL3*, *IFNL2*, and *CXCL1* each had a degree score of 11. *CCN1* and *IL19* scored 10.

The topology of the hub gene set reflects two converging response programs to 5-FU treatment. The first is an inflammatory–cytokine axis centered on *IL6* (degree = 30), which drives *STAT3*-mediated survival signaling and is a well-established mediator of 5-FU chemoresistance in CRC^80^. The co-enrichment of *CXCL1*, *IL19*, *IFNL2*, and *IFNL3* around this hub suggests that 5-FU treatment activates broad intercellular immune signaling rather than a single cytokine pathway, a finding that may have implications for the inflammatory side effects observed clinically. The second program is a stress-transcription axis comprising *MYC*, *EGR1*, and *ATF3*, all of which are immediate-early response genes. Their simultaneous upregulation points to a coordinated transcriptional response to genotoxic stress: *EGR1* and *ATF3* can independently drive *p21* and *PTEN* expression to enforce cell cycle arrest^81,82^, while *MYC*, paradoxically upregulated, likely reflects the competing proliferative pressure that determines whether cells commit to apoptosis or adaptation. The presence of *BMP4* at the periphery of this cluster is notable given its reported role in sensitizing CRC stem cells to 5-FU through differentiation induction^77^.

**Table 7:** Top 10 hub genes ranked by degree score from the PPI network (CytoHubba analysis). Higher scores reflect greater connectivity and potential regulatory importance.

Rank	Name	Score
1	*IL6*	30
2	*MYC*	16
3	*EGR1*	14
4	*BMP4*	13
5	*ATF3*	12
6	*IFNL3*	11
6	*IFNL2*	11
6	*CXCL1*	11
9	*CCN1*	10
9	*IL19*	10

#### Functional enrichment analysis

**Fig. 14:**
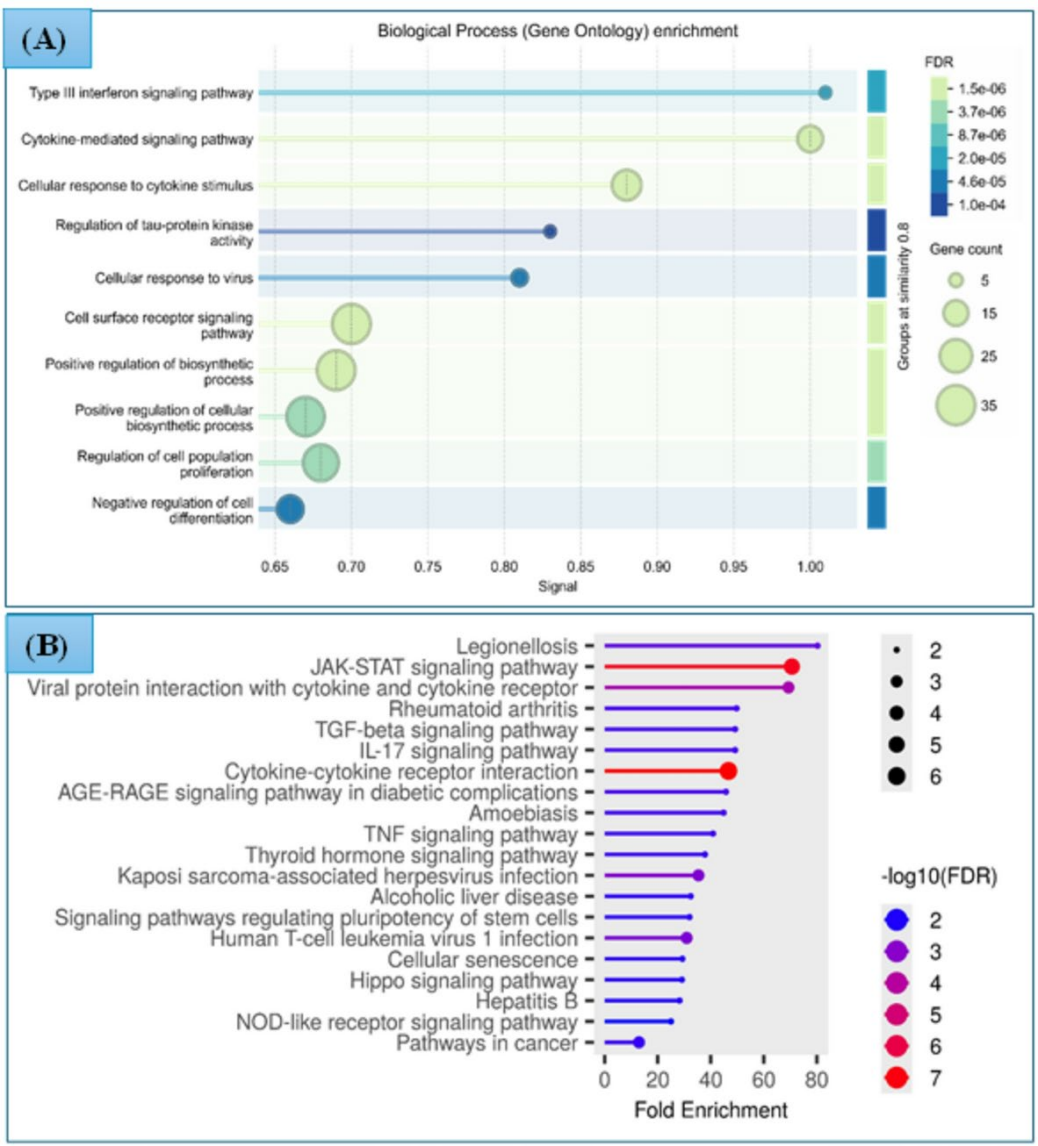
Functional enrichment analysis before and after hub gene selection. (**A**) GO biological process enrichment of all dysregulated genes (STRING). (**B**) Pathway enrichment of the top 10 hub genes with key terms including *JAK*–*STAT* signaling and cytokine–cytokine receptor interaction. Bubble size = gene count; color = FDR.

The functional enrichment analysis showed overrepresentation of differentially expressed genes in immune response, cell signaling, and cell proliferation–related biological processes (Fig. 14A). The most enriched term was the cytokine-mediated signaling pathway (FDR = 8.77 × 10⁻⁵). This term involved seven hub genes: *EGR1*, *IFNL2*, *IL19*, *CXCL1*, *IL6*, *IFNL3*, and *MYC* (Table 8). These genes mediate intercellular communication during inflammatory and immune responses.

The co-enrichment of IL6, MYC, and EGR1 within the cytokine-mediated signaling pathway term reveals a tension that is central to understanding 5-FU pharmacology. *IL6*/*GP130*–*STAT3* signaling is one of the best-characterized drivers of 5-FU resistance in CRC: persistent *IL6* activity maintains anti-apoptotic gene expression and reduces drug uptake^83^. Yet *EGR1*, induced here by 5-FU-mediated DNA damage, counteracts this by transcriptionally activating *p21* and *PTEN* independently of *p53* status^84,85^. The simultaneous upregulation of *MYC* alongside *EGR1* in this dataset therefore represents competing outputs, *MYC* sustaining a proliferative programmed while *EGR1* attempts to enforce arrest, and the balance between these signals may determine whether individual cells undergo apoptosis or develop adaptive resistance. This interpretation aligns with the in vitro gene expression data showing that the ternary nanocarrier, by delivering 5-FU more gradually, may shift this balance toward apoptosis by avoiding the acute *IL6*-mediated survival response triggered by free drug.

The interferon family members IFNL2 and *IFNL3* also emerged as modulators. Both activate the JAK–STAT pathway (*STAT1*, *STAT2*, and *STAT3*) and influence antiproliferative responses similar to *IFN‑α* and *IFN‑γ*^86^. Amplification of *IFNL2*/*IFNL3* genes has been reported in poor‑prognosis cancers, correlating with *TP53* mutations, reduced DNA methylation, and altered immune checkpoint activity^87,88^. *CXCL1* was upregulated in response to 5‑FU, a change linked to inflammation and adverse effects including diarrhea during therapy^89^. *CCN1* is a matricellular protein frequently overexpressed in colorectal cancers and associated with poor prognosis, tumor progression, and metastasis^77^. *IL19* is a cytokine that may modulate inflammatory responses during chemotherapy, similar to IL6^90^.

Beyond cytokine signaling, other enriched biological processes were observed. Terms included “cellular response to organic substances” (FDR = 3.3 × 10^− 4^) and “response to chemical” (FDR = 5.3 × 10^− 4^). Enrichment of “positive regulation of cell death” and “positive regulation of apoptotic process” appeared, consistent with the cytotoxic effects of 5‑FU observed in vitro.

Developmental processes, including kidney development and metanephros development, were also enriched. These involved genes such as *EGR1*, *BMP4*, and *MYC*. Although these terms are classically linked to organogenesis, the implicated genes are also involved in tissue remodeling and repair^91^. *BMP4* promotes differentiation and apoptosis of colorectal cancer stem cells and enhances the antitumor effects of 5‑FU^92^. *ATF3* is upregulated by 5‑FU and plays roles in the DNA damage response, cell cycle control, and apoptosis^81^. At the pathway level, enrichment in *MAPK* cascade regulation and JAK–STAT receptor signaling appeared (Fig. 14B). Additional enrichment of immune‑related processes such as defense response to viruses and chemotaxis was observed^93^.

**Table 8:** GO biological process enrichment of dysregulated genes. Key terms include cytokine signaling, stress responses, apoptosis, immune processes, and signal transduction, indicating treatment effects on immune modulation and communication.

Description	Fdr	Preferred Names	Number of genes
Cytokine-mediated signaling pathway	8.77E-05	*EGR1*,*IFNL2*,*IL19*,*CXCL1*,*IL6*,*IFNL3*,*MYC*	7
Cellular response to organic substance	0.00033	*EGR1*,*BMP4*,*IFNL2*,*IL19*,*ATF3*,*CXCL1*,*IL6*,*IFNL3*,*MYC*	9
Regulation of cell proliferation involved in kidney development	0.00033	*EGR1*,*BMP4*,*MYC*	3
Response to chemical	0.00053	*EGR1*,*BMP4*,*IFNL2*,*IL19*,*ATF3*,*CXCL1*,*IL6*,*CYR61*,*IFNL3*,*MYC*	10
Regulation of metanephros development	0.00053	*EGR1*,*BMP4*,*MYC*	3
Positive regulation of cell death	6.00E-04	*EGR1*,*BMP4*,*ATF3*,*IL6*,*CYR61*,*MYC*	6
Signal transduction	0.0011	*EGR1*,*BMP4*,*IFNL2*,*IL19*,*ATF3*,*CXCL1*,*IL6*,*CYR61*,*IFNL3*,*MYC*	10
Regulation of molecular function	0.0011	*EGR1*,*BMP4*,*IFNL2*,*IL19*,*ATF3*,*CXCL1*,*IL6*,*CYR61*,*IFNL3*,*MYC*	10
Positive regulation of kidney development	0.0012	*EGR1*,*BMP4*,*MYC*	3
Cell surface receptor signaling pathway	0.0013	*EGR1*,*BMP4*,*IFNL2*,*IL19*,*CXCL1*,*IL6*,*IFNL3*,*MYC*	8
Response to external stimulus	0.0013	*EGR1*,*BMP4*,*IFNL2*,*ATF3*,*CXCL1*,*IL6*,*CYR61*,*IFNL3*	8
Positive regulation of cell population proliferation	0.0015	*EGR1*,*BMP4*,*ATF3*,*IL6*,*CYR61*,*MYC*	6
Regulation of cell population proliferation	0.0019	*EGR1*,*BMP4*,*ATF3*,*CXCL1*,*IL6*,*CYR61*,*MYC*	7
Positive regulation of osteoblast differentiation	0.0023	*BMP4*,*IL6*,*CYR61*	3
Positive regulation of apoptotic process	0.0057	*BMP4*,*ATF3*,*IL6*,*CYR61*,*MYC*	5
Positive regulation of cell proliferation involved in kidney development	0.0057	*EGR1*,*MYC*	2
Positive regulation of transcription by RNA polymerase II	0.0059	*EGR1*,*BMP4*,*ATF3*,*IL6*,*CYR61*,*MYC*	6
Regulation of glomerular mesangial cell proliferation	0.0061	*EGR1*,*BMP4*	2
Regulation of MAPK cascade	0.007	*BMP4*,*ATF3*,*IL6*,*CYR61*,*MYC*	5
Cell proliferation involved in kidney development	0.007	*EGR1*,*BMP4*	2
Positive regulation of production of miRNAs involved in gene silencing by miRNA	0.0076	*BMP4*,*IL6*	2
Positive regulation of response to stimulus	0.0096	*BMP4*,*IFNL2*,*ATF3*,*IL6*,*CYR61*,*IFNL3*,*MYC*	7
Regulation of protein phosphorylation	0.0106	*EGR1*,*BMP4*,*ATF3*,*IL6*,*CYR61*,*MYC*	6
Positive regulation of metanephros development	0.0106	*EGR1*,*MYC*	2
Response to stress	0.0113	*EGR1*,*IFNL2*,*ATF3*,*CXCL1*,*IL6*,*CYR61*,*IFNL3*,*MYC*	8
Nephron development	0.0113	*EGR1*,*BMP4*,*MYC*	3
Regulation of apoptotic process	0.0131	*EGR1*,*BMP4*,*ATF3*,*IL6*,*CYR61*,*MYC*	6
Immune system process	0.0141	*EGR1*,*BMP4*,*IFNL2*,*IL19*,*CXCL1*,*IL6*,*IFNL3*	7
Immune response	0.0141	*EGR1*,*IFNL2*,*IL19*,*CXCL1*,*IL6*,*IFNL3*	6
Glomerulus vasculature development	0.0143	*EGR1*,*BMP4*	2
Negative regulation of MAPK cascade	0.0186	*BMP4*,*ATF3*,*MYC*	3
Positive regulation of molecular function	0.023	*EGR1*,*BMP4*,*CXCL1*,*IL6*,*CYR61*,*MYC*	6
Chemotaxis	0.0241	*BMP4*,*CXCL1*,*IL6*,*CYR61*	4
Regulation of response to stimulus	0.0241	*EGR1*,*BMP4*,*IFNL2*,*ATF3*,*IL6*,*CYR61*,*IFNL3*,*MYC*	8
Positive regulation of cartilage development	0.0254	*BMP4*,*CYR61*	2
Regulation of mesenchymal cell proliferation	0.0262	*BMP4*,*MYC*	2
Defense response to virus	0.0264	*IFNL2*,*IL6*,*IFNL3*	3
Positive regulation of BMP signaling pathway	0.0265	*BMP4*,*CYR61*	2
Regulation of collagen biosynthetic process	0.0303	*BMP4*,*IL6*	2
Connective tissue development	0.0303	*EGR1*,*BMP4*,*CYR61*	3
Receptor signaling pathway via JAK-STAT	0.0311	*IFNL2*,*IFNL3*	2
Endocrine pancreas development	0.0318	*BMP4*,*IL6*	2
Organ or tissue specific immune response	0.0325	*IFNL2*,*IL6*	2
Positive regulation of bone mineralization	0.0325	*BMP4*,*CYR61*	2
Response to other organism	0.0335	*EGR1*,*IFNL2*,*CXCL1*,*IL6*,*IFNL3*	5
Negative regulation of signal transduction	0.0341	*EGR1*,*BMP4*,*ATF3*,*IL6*,*MYC*	5
Branching involved in ureteric bud morphogenesis	0.036	*BMP4*,*MYC*	2
Defense response	0.0361	*EGR1*,*IFNL2*,*CXCL1*,*IL6*,*IFNL3*	5
Positive regulation of epithelial to mesenchymal transition	0.0394	*BMP4*,*IL6*	2
Negative regulation of cell population proliferation	0.0405	*BMP4*,*CXCL1*,*IL6*,*MYC*	4
Positive regulation of transferase activity	0.0416	*EGR1*,*BMP4*,*CYR61*,*MYC*	4
Skeletal muscle cell differentiation	0.0432	*EGR1*,*ATF3*	2
Heart valve morphogenesis	0.0442	*BMP4*,*CYR61*	2
Striated muscle tissue development	0.0442	*EGR1*,*BMP4*,*ATF3*	3
Positive regulation of developmental process	0.0442	*EGR1*,*BMP4*,*IL6*,*CYR61*,*MYC*	5
Regulation of pri-miRNA transcription by RNA polymerase II	0.0442	*EGR1*,*BMP4*	2
Negative regulation of RNA metabolic process	0.0456	*EGR1*,*BMP4*,*ATF3*,*IL6*,*MYC*	5
Regulation of ERK1 and ERK2 cascade	0.0456	*BMP4*,*ATF3*,*CYR61*	3

These findings indicate that 5‑FU induces transcriptional changes across immune activation, apoptosis, stress adaptation, and developmental pathways. This interplay of signaling networks may underlie both the therapeutic efficacy and the toxicological effects of 5‑FU. Cytokines including IL6 and *IL19* appear as modulators of treatment response. Further studies are needed to elucidate their roles in chemoresistance and sensitivity in colorectal cancer.

Taken together, the in vitro and in silico data suggest a coherent mechanistic picture. At the cellular level, the pH-responsive release of 5-FU from the ternary nanocarrier produces sustained intracellular drug concentrations sufficient to activate the *P53*–*Caspase-3* axis and suppress *BCL2*, without triggering the acute survival response, reflected in paradoxical *BCL2* upregulation, that accompanies free drug delivery. At the network level, the in silico analysis identifies *IL6*–*STAT3* signaling and the *MYC*/*EGR1* stress-transcription axis as the primary nodes through which 5-FU reshapes the CRC transcriptome. The stronger modulation of apoptotic and angiogenic markers observed with the nanocarrier formulation in vitro is consistent with a delivery profile that sustains drug exposure long enough to overcome the *IL6*-mediated chemoresistance mechanism identified in the network analysis. This convergence between the two approaches strengthens confidence in both datasets and provides a rationale for prioritizing IL6 pathway co-targeting in future formulation studies.

## Conclusion

A ternary drug delivery system was developed by physically assembling chitosan and β‑cyclodextrin into a pseudopolyrotaxane complex, followed by incorporation of 5‑FU via solvent evaporation to form a solid dispersion. The obtained nanocomposite averaged 187 nm in diameter, carried a positive zeta potential (+ 32.5 mV), and released 5‑FU faster at pH 5.5 than at pH 7.4. In HCT‑116 cells, the ternary formulation had an IC_50_ of 16.5 µM, about 2.6‑fold lower than free 5‑FU. This was accompanied by stronger increase in *P53* and *Caspase‑3* expression and more pronounced decrease in *BCL2* and *VEGF* expression. An in silico analysis of 5‑FU transcriptional responses identified *IL6*, *MYC*, *EGR1*, and *ATF3* as hub genes linked to apoptosis and cell‑cycle control. These results indicate that embedding 5‑FU in a chitosan/β‑cyclodextrin matrix improves its anticancer activity against colorectal cancer cells, likely through better solubility, sustained release, and enhanced engagement of apoptotic pathways. In vivo pharmacokinetic and efficacy studies should be performed to assess the formulation’s behavior in animal models. Long‑term colloidal and chemical stability under storage conditions requires further evaluation. The mechanistic roles of the identified hub genes (*IL6*, *MYC*, *EGR1*, *ATF3*) in nanocarrier‑mediated 5‑FU delivery could be validated through targeted gene knockdown or inhibition experiments. Finally, the physical assembly method used here may be applicable to other hydrophilic chemotherapeutics with poor bioavailability.

## Study Limitations

The in silico analysis was conducted exclusively for 5-fluorouracil due to the absence of transcriptomic datasets for chitosan and β-cyclodextrin or their complexes. Therefore, the identified hub genes and enriched pathways reflect the molecular response to 5-FU itself, not to the nanocarrier components. The in silico findings are presented as correlative systems-level context for 5-FU pharmacology, not as direct evidence for nanocarrier-specific effects. Further studies are warranted, including in vivo pharmacokinetic and efficacy evaluations, long-term stability assessment, and mechanistic validation of the proposed molecular pathways.

## Data Availability

All data supporting the findings of this study are included within the article. Additional data are available from the corresponding author upon request.
